# Synthesis, Chemical Characterization and Multiscale Biological Evaluation of a Dimeric-cRGD Peptide for Targeted Imaging of *α*_*V*_*β*_3_ Integrin Activity

**DOI:** 10.1038/s41598-017-03224-8

**Published:** 2017-06-09

**Authors:** Jamila Hedhli, Andrzej Czerwinski, Matthew Schuelke, Agata Płoska, Paweł Sowinski, Lukas La Hood, Spencer B. Mamer, John A. Cole, Paulina Czaplewska, Maciej Banach, Iwona T. Dobrucki, Leszek Kalinowski, Princess Imoukhuede, Lawrence W. Dobrucki

**Affiliations:** 1 Beckman Institute for Advanced Science and Technology, Urbana, IL USA; 2 Department of Bioengineering, University of Illinois at Urbana-Champaign, Urbana, IL USA; 3 Peptides International Inc., Louisville, KY USA; 4 Department of Laboratory Diagnostics, Medical University of Gdansk, Gdansk, Poland; 5 NMR Laboratory, Faculty of Chemistry, Gdansk University of Technology, Gdansk, Poland; 6 Department of Physics, University of Illinois at Urbana-Champaign, Urbana, IL USA; 7 Intercollegiate Faculty of Biotechnology of the University of Gdansk and Medical University of Gdansk, Gdansk, Poland; 8 Department of Hypertension, Medical University of Lodz, Lodz, Poland

## Abstract

Cyclic peptides containing the Arg-Gly-Asp (RGD) sequence have been shown to specifically bind the angiogenesis biomarker *α*_*V*_ *β*_3_ integrin. We report the synthesis, chemical characterization, and biological evaluation of two novel dimeric cyclic RGD-based molecular probes for the targeted imaging of *α*_*V*_ *β*_3_ activity (a radiolabeled version, ^64^Cu-NOTA-PEG_4_-cRGD_2_, for PET imaging, and a fluorescent version, FITC-PEG_4_-cRGD_2_, for *in vitro* work). We investigated the performance of this probe at the receptor, cell, organ, and whole-body levels, including its use to detect diabetes associated impairment of ischemia-induced myocardial angiogenesis. Both versions of the probe were found to be stable, demonstrated fast receptor association constants, and showed high specificity for *α*_*V*_ *β*_3_ in HUVECs (*K*_*d*_ ~ 35 nM). Dynamic PET-CT imaging indicated rapid blood clearance via kidney filtration, and accumulation within *α*_*V*_ *β*_3_-positive infarcted myocardium. ^64^Cu-NOTA-PEG_4_-cRGD_2_ demonstrated a favorable biodistribution, slow washout, and excellent performance with respect to the quality of the PET-CT images obtained. Importantly, the ratio of probe uptake in infarcted heart tissue compared to normal tissue was significantly higher in non-diabetic rats than in diabetic ones. Overall, our probes are promising agents for non-invasive quantitative imaging of *α*_*V*_ *β*_3_ expression, both *in vitro* and *in vivo*.

## Introduction

The development of capillaries from preexisting vessels to form new vasculature, termed as angiogenesis, is tightly controlled by a delicate balance of pro- and anti-angiogenic factors. The main stimulators of blood vessel formation are vascular endothelial growth factor (VEGF) and basic fibroblast growth factor (bFGF), while endogenous inhibitors include thrombospondin, and angiostatin^1^. The process of creating new blood vessels is necessary for physiological function, but is also a hallmark of many pathological disorders such as inflammation, ischemic heart disease, and cancer.

Over the last two decades, significant research efforts have been directed at developing novel targeted imaging techniques for the non-invasive assessment of angiogenesis as a tool for early diagnosis, prognostication, and monitoring of novel individualized therapeutic interventions focused on the modulation of the angiogenic process^2^. These efforts resulted in the discovery of numerous potential imaging targets including integrins, which play an important role in the regulation of angiogenesis^3^. Found in species ranging from sponges to mammals, integrins are heterodimer transmembrane glycoproteins consisting of *α* (150–180 kD) and *β* (90 kD) subunits. The cell surface receptor is composed of three parts: a large ectodomain, a single transmembrane domain, and a short cytoplasmic tail. Integrins, when activated by an intracellular ligand (*e*.*g*. talin or kindlin), become capable of binding specific extracellular molecules in a unique combination of “inside-out” and “outside-in” signaling. In response, the integrins elicit a cascade of biochemical events that modulate gene expression, and regulate cytoskeleton organization^4–6^.

Significant effort has been expended exploring the clinical utility of imaging *α*_*V*_ *β*_3_^7, 8^ expression associated with tumor angiogenesis and metastatic dissemination^9, 10^. For example, radiolabeled integrin peptide ligands with a pentapeptide motif Arg-Gly-Asp-Phe-Lys (RGDfK) have been used as tools in research aimed at developing radiotracers for clinical applications^11, 12^. Numerous linear and cyclic RGD peptides based (to a greater or lesser extent) on the RGDfK motif, in combination with a variety of pharmacokinetic modifiers, prosthetic groups or bifunctional chelators and radionuclides have been reported and used as diagnostic probes in animal studies and clinical investigations in the areas of oncology^13–23^ and cardiology^24–27^.

Multivalent interactions have been shown to play a key role in many biological processes^28^. This phenomenon was behind the rationale for the design of multimeric cRGD ligands. A series of such peptides labeled with ^18^F, ^64^Cu and ^68^Ga for PET or ^99*m*^Tc for SPECT imaging have been reported^29–33^. They demonstrated higher receptor binding affinity *in vitro* and better tumor retention *in vivo* when compared to their corresponding monomeric counterparts. PEG and other types of linkers have been employed as pharmacokinetic modifiers, resulting in significantly increased tumor uptake and enhanced clearance from noncancerous organs^34–38^. Comparisons of different RGD-based tracers have demonstrated that increasing the peptide multiplicity can greatly improve affinity, but above a certain threshold, highly multimeric compounds exhibit non-specific organ uptake that can limit their applicability as imaging agents. This led to the conclusion that the dimeric subclass of the RGD peptides is an optimal choice for the development of imaging agents for clinical applications^15, 23, 39^. The SPECT tracer ^99*m*^Tc-3PRGD_2_^34, 40–43^ as well as several RGD-based PET radiotracers^44–48^, have already entered clinical investigation stage^12^.

In the design of our new *α*_*V*_ *β*_3_ targeted probe we integrated several structural elements known for their positive impact on pharmacokinetic properties including PEG moieties and 1,2,3-triazole residues^49^. Here we report the synthesis and chemical characterization of *α*_*V*_ *β*_3_ integrin targeted ^64^Cu-labeled dimeric-cRGD probe (designed for *in vivo* PET imaging) and its FITC-labeled fluorescent analogue (designed for *in vitro* imaging with single-cell resolution). Using established *in vitro* and *in vivo* models we evaluated its efficacy as an imaging agent at the receptor, single cell, organ, and whole-body levels with the emphasis on quantitative assessment of *α*_*V*_ *β*_3_ integrin expression in diabetic and non-diabetic rats following myocardial infarction (MI).

## Results

### Synthesis and Chemical Characterization

**Figure 1:**
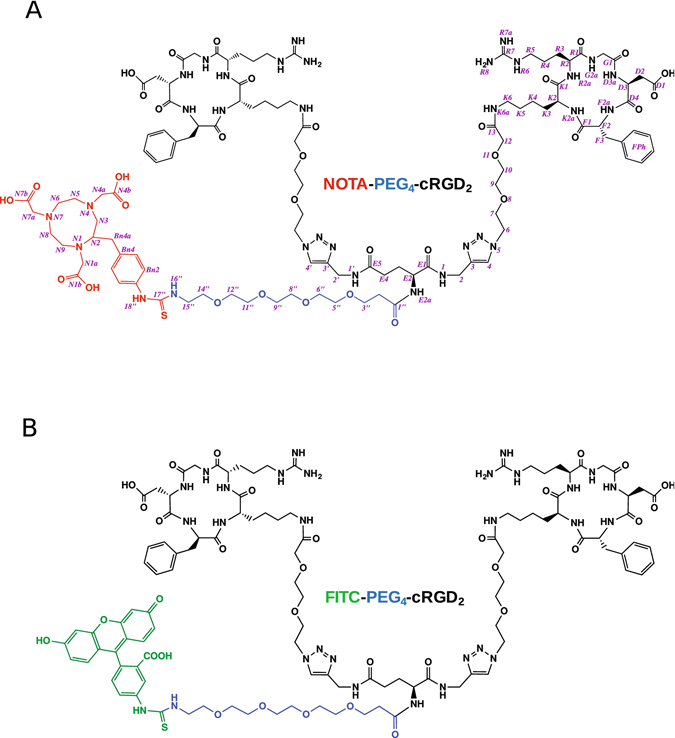
Chemical structures of (**A**) NOTA-PEG_4_-cRGD_2_ and (**B**) FITC-PEG_4_-cRGD_2_.

The synthesis (Supplementary Figure S1) began with coupling of N_3_-PEG_2_-OH to a selectively protected cyclopentapeptide, yielding azidopegylated protected peptide (**1**). An important aspect of our design was related to the dimerization method. Dimeric peptides have usually been prepared by an acylation reaction using cRGD monomers and N-protected glutamic acid active esters, e.g. Boc-Glu(OSu)_2_ as the substrates^30, 31, 50^. In our case, the dimerization was achieved by employing Boc-glutamic acid bis-propargyl amide as an alkyne substrate to bridge two RGD pegylated cyclopentapeptides via the copper (I)-catalyzed 1,3-dipolar Huisgen cycloaddition, yielding the protected product (**2**). This is only the second synthesis of the cRGD dimer, after galacto-RGD_2_, based on the aforementioned click reaction protocol^38^. Total deprotection of (**2**), followed by the PEGylation reaction using Boc-PEG_4_-OSu, and Boc-group removal yielded compound (**5**). After RP-HPLC preparative purification, product (**5**) was used as the substrate for the synthesis of both tracers, ^64^Cu-NOTA-PEG_4_-cRGD_2_ (**7**) and fluorescent analogue FITC-PEG_4_-cRGD_2_ (**8**). Briefly, product (**7**) was prepared in the reaction of (**5**) with p-SCN-Bn-NOTA, followed by the radiolabeling of NOTA-chelated compound (**6**). Reaction of (**5**) with fluorescein-5- isothiocyanate yielded the fluorescent product (**8**). Based on the ^1^H, ^13^C, DQF-COSY, TOCSY, NOESY NMR and H/C correlation spectra (gHSQC and gHMBC, Supplementary Figures S2–S8), a full structural analysis of NOTA-PEG_4_-cRGD_2_ (**6**) was performed, including assignment of all proton and carbon resonances (Supplementary Tables S2 and S3). To confirm the successful synthesis and measure masses of NOTA-PEG_4_-cRGD_2_ (**6**) and FITC-PEG_4_-cRGD_2_ (**8**) see (Fig. 1), high-resolution MS spectra for pure products were registered in direct infusion mode. For product (**6**) the three- and four-fold charged molecular ions were identified on ESI spectrum (Supplementary Figure S8).

### Dimeric cRGD Probe Binds Preferentially to the *α*_*V*_*β*_3_ Integrin Receptor

#### Receptor binding kinetics

We investigated the receptor binding kinetics of both the FITC- and ^64^Cu-labeled tracers using surface plasmon resonance (SPR). As described in Methods and Supporting Information sections, initial pre-concentration studies were focused on adjusting the pH to optimize protein immobilization. We then evaluated the association and dissociation rates of our probes to the immobilized *α*_*V*_ *β*_3_ receptors and compared these values with those of a commercially available monomeric probe, cRGDyK.

**Figure 2:**
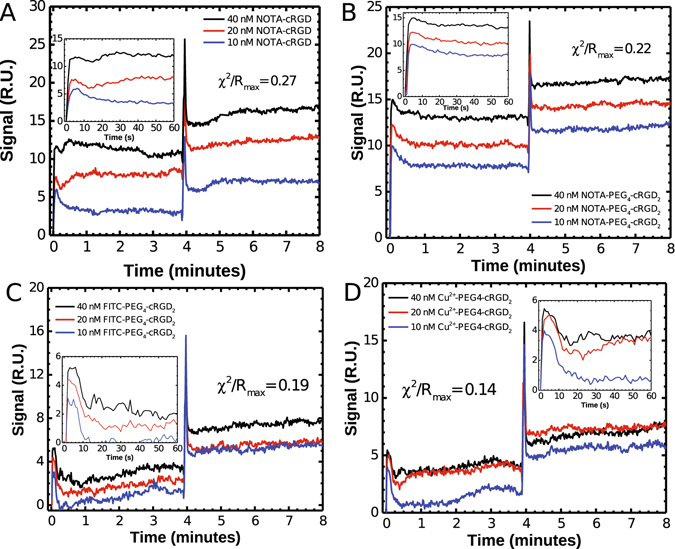
SPR sensograms depicting binding between cRGD ligands and *α*_*V*_ *β*_3_ integrin receptor. BIAcore 3000 kinetics studies of interactions between immobilized integrin *α*_*V*_ *β*_3_ receptor and (**A**) monomeric cyclic RGD probe (NOTA-cRGD), (**B**) dimeric cyclic RGD (NOTA-PEG_4_-cRGD_2_) probe, (**C**) cRGD_2_ conjugated with FITC (FITC-PEG_4_-cRGD_2_), and (**D**) NOTA-PEG_4_-cRGD_2_ labeled with non-radioactive Cu^2+^. Kinetic studies were performed at a 30 *μ* L/min flow rate, with a 4 min association followed by a 10 min dissociation period.

The SPR sensograms associated with each probe demonstrated significant accumulation upon initial injection (Fig. 2). Their association phases were characterized by a sharp signal increase at the start of the injection, followed by a slow decrease following the injection. We observed that unlabeled dimeric probe bound *α*_*V*_ *β*_3_ with an affinity (*K*_*d*_) approximately 50 times greater than the monomeric cRGD (Table 1), which was paralleled by a larger magnitude response *R*_*max*_ = 10 R.U. (Fig. 2B) compared to ~5 R.U. (Fig. 2A). Both labeled versions of our probe showed only marginally reduced binding affinity for *α*_*V*_ *β*_3_ relative to the unlabeled version, but they both outperformed the monomeric cRGD-based probe.

**Table 1:** Summary of binding affinity and kinetic rates measured between cRGD-based monomeric and dimeric probes and immobilized integrin *α*_*V*_ *β*_3_ receptor using a surface plasmon resonance-based approach.

Probe	*K*_*on*_ (M^−1^ s^−1^)	*K*_*off*_ (s^−1^)	*K*_*D*_ (pM)	*χ* ^2^/*Rmax*
NOTA-cRGD	2.7 × 10^5^	2.6 × 10^−6^	9.6	0.26
NOTA-PEG_4_-cRGD_2_	1.1 × 10^7^	2.1 × 10^−6^	1.9 × 10^−1^	0.22
FITC-PEG_4_-cRGD_2_	7.6 × 10^5^	6.6 × 10^−6^	8.6	0.19
^64^Cu-NOTA-PEG_4_-cRGD_2_	7.1 × 10^5^	1.1 × 10^−6^	1.5	0.14

Kinetic constants were obtained by performing global kinetic analysis using the BIAevaluation software across several kinetic binding sensograms for each ligand-receptor pair.

#### Binding affinity in HUVECs

**Figure 3:**
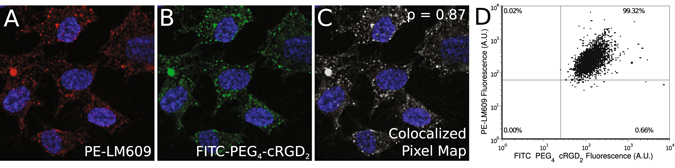
Colocalization between phycoerythrin-labeled anti- *α*_*V*_ *β*_3_ integrin primary antibody (PE-LM609) and FITC-labeled PEG_4_-cRGD_2_ probe. Human umbilical vein endothelial cells (HUVEC) were grown to confluency and incubated with both PE-LM609 and FITC-PEG_4_-cRGD_2_. Fluorescence microscopy images were acquired in DAPI/PE (**A**) and DAPI/FITC (**B**) channels and were superimposed to create colocalized pixel map (**C**) to calculate Pearson’s coefficient. Flow cytometric analysis of HUVEC co-incubated with PE-LM609 and FITC-PEG_4_-cRGD_2_ demonstrated a very high degree of colocalization between integrin *α*_*V*_ *β*_3_ and FITC-PEG_4_-cRGD_2_ probe (**D**).

**Figure 4:**
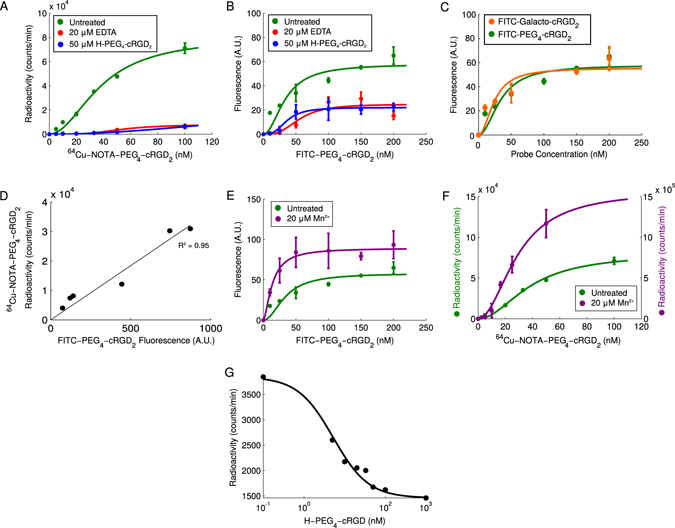
Binding kinetics of FITC and ^64^Cu labeled cRGD _2_ probes. (**A**) Radioactivity of confluent HUVEC cells incubated with varying concentrations of ^64^Cu-NOTA-PEG_4_-cRGD_2_ (green) and with either 20 *μ*M EDTA (red) or 50 *μ*M of H-PEG_4_-cRGD_2_ (blue). (**B**) Fluorescence of the FITC-PEG_4_-cRGD_2_ (green) with either 20 *μ*M EDTA (red) or 50 *μ*M of H-PEG_4_-cRGD_2_ (blue). (**C**) Comparison of the fluorescence of the FITC-PEG_4_-cRGD_2_ (green) with FITC-Galacto-cRGD_2_ (orange). (**D**) Correlation between FITC and Cu^64^ labeled cRGD_2_ probes bound to HUVECs. (**E**) The fluorescence of HUVEC cells incubated with varying concentrations of FITC-PEG_4_-cRGD_2_ (green) and co-incubated with 20 *μ*M of Mn^2+^ (purple). (**F**) Radioactivity of confluent HUVEC cells incubated with varying concentrations of ^64^Cu-NOTA-PEG_4_-cRGD_2_ (green) and co-incubated with 20 *μ*M of Mn^2+^ (purple). (**G**) Competition binding between ^64^Cu-NOTA-PEG_4_-cRGD_2_ (50 nM) and increasing concentrations of unlabeled H-PEG_4_-cRGD_2_ in HUVEC cells.

We next studied the cellular binding properties of both (**7**) and (**8**). For our studies we used human umbilical vein endothelial cells (HUVECs), which are known to constitutively express *α*_*V*_ *β*_3_ integrin. HUVECs were first treated with (**8**) and co-incubated with the anti- *α*_*V*_ *β*_3_ antibody. Subsequent two-channel confocal fluorescent microscopic images (Fig. 3A–C) demonstrated a significant degree of overlap between the fluorescein and phycoerythrin signals (Pearson’s coefficient *ρ* = 0.87), which indicates a strong co-localization of (**8**) with *α*_*V*_ *β*_3_ integrin receptor. The specificity of (**8**) was further assessed on a single cell level using flow cytometry. Figure 3D shows a quadrant plot of HUVECs treated with (**8**) and co-incubated with the anti- *α*_*V*_ *β*_3_ antibody, which showed that (**8**) successfully bound over 99% of HUVECs expressing *α*_*V*_ *β*_3_ integrin. To quantitatively evaluate binding parameters, cultured HUVECs were incubated with various concentrations of (**7**) and (**8**) followed by cellular binding assay with gamma well counting and flow cytometry. The observed radioactivity and fluorescence intensity was found to increase with probe concentration and fit well to a Hill-type function (Fig. 4A and B). This allowed us to estimate the dissociation constant (*K*_*d*_) at 38.27 nM and 33.85 nM for the (**8**) and (**7**), respectively. We also compared our (**8**) to a commercially available probe FITC-Galacto-cRGD_2_ and found similar binding kinetics (Fig. 4C). Both (**7**) and (**8**) also showed strong correlation (*R* ^2^ = 0.95) over the range of concentrations used in the cellular binding experiments (Fig. 4D).

#### Binding affinity in the presence of metal ions

We incubated HUVECs with either 20 *μ*M of Mn^2+^ or EDTA and measured the B_*max*_ and K_*d*_ with flow cytometry and gamma well counting. The maximum fraction of bound receptors was found to decrease in the presence of EDTA (Fig. 4) for both probes. Interestingly, the ^64^Cu-labeled probe showed a significantly more pronounced decrease in B_*max*_, which we attribute to the scavenging of ^64^Cu by EDTA. The dissociation constants for both the ^64^Cu- and FITC-labeled probes in the presence of EDTA were measured to be approximately 55 nM, indicating a similar effect on the binding kinetics. In the presence of Mn^2+^ the probes’ binding was found to increase by 78% when compared to HUVECs incubated without Mn^2+^ (Fig. 4E and F). Likewise, the dissociation constant in the presence of Mn^2+^ was considerably lower reaching 13.8 nM.

#### Competitive binding

Finally, we studied competitive *α*_*V*_ *β*_3_ integrin binding properties for our dimeric-cRGD probes. HUVECs were pre-treated with an excess of unlabeled NOTA-PEG_4_-cRGD_2_ (50 *μ*M) followed by incubation with various concentrations of (**7**) or (**8**). This resulted in a noticeable decrease in *B*_*max*_ and an increase in the *K*_*d*_ (188 nM) compared to untreated cells (Fig. 4A,B). To determine IC _50_ values, HUVEC were treated with a fixed concentration of the probe (50 nM) and incubated with a range of unlabeled NOTA-PEG_4_-cRGD_2_ concentrations. The results of these experiments were fit to a model of homologous inhibition and yielded an approximate IC _50_ of 5.1 nM (Fig. 4G)^51^.

### Pharmacokinetics, Biodistribution, and PET Imaging

**Figure 5:**
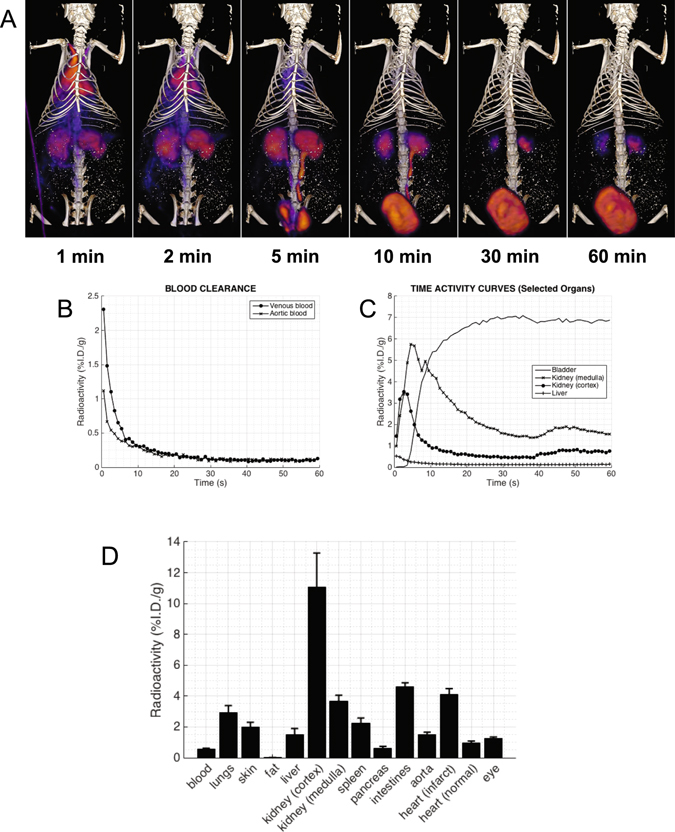
Dynamic PET-CT images (**A**) were used to plot the blood clearance (**B**) and time activity curves (TAC) of ^64^Cu-NOTA-PEG_4_-cRGD_2_ in selected organs (**C**). (**D**) Biodistribution of ^64^Cu-NOTA-PEG_4_-cRGD_2_ in selected organs at 90 min post-injection in Lewis rats subjected to myocardial infarction induced by surgical ligation of LAD. Results are expressed in percentage of injected dose per gram tissue (%I.D./g). These results suggest a rapid blood clearance through renal filtration and very low non-specific uptake in other critical organs.

Dynamic *in vivo* PET-CT imaging of *α*_*V*_ *β*_3_ integrin activity (Fig. 5A) demonstrated favorable initial biodistribution and clearance kinetics for (**7**). Clearance kinetics from critical organs during the first 60 min after injection was quantified by placing volumes of interest (VOIs) on the dynamic co-registered PET-CT image sets. The radioactivity within these VOIs was expressed as percent injected dose per gram tissue (%I.D./g). The representative blood and tissue activity curves are shown in (Fig. 5B and C). After intravascular injection, (**7**) cleared rapidly from the blood, accumulated primarily in the kidneys, and was excreted in the urine, which was retained in the bladder as early as 15 min after injection.

Figure 5D shows the biodistribution data of (**7**) at 90 min after intravascular injection into control rats and animals subjected to the LAD ligation. As expected, the highest uptake was observed in the kidneys due to renal filtration being the dominate excretion route (11.08 ± 2.20 and 3.66 ± 0.40 %I.D./g for cortex and medulla, respectively). Relatively high uptake was also observed in the intestines (4.62 ± 0.24), lungs (2.95 ± 0.43), and the spleen (2.25 ± 0.33).

To assess *in vivo* stability of (**7**) we performed metabolism studies at the end of the dynamic PET-CT acquisitions. Figure SI 9A,B shows representative HPLC radiochromatograms of (**7**) prepared immediately before injection into the animal, and non-targeted ^64^Cu-acetate in ammonium acetate buffer, respectively. These studies allowed us to determine the retention times for (**7**) and unbound ^64^Cu, which were further used to demonstrate that more than 90% of (**7**) remained intact in the urine (see Figure SI 9C) while there was no detectable activity in the feces (see Figure SI 9D).

**Figure 6:**
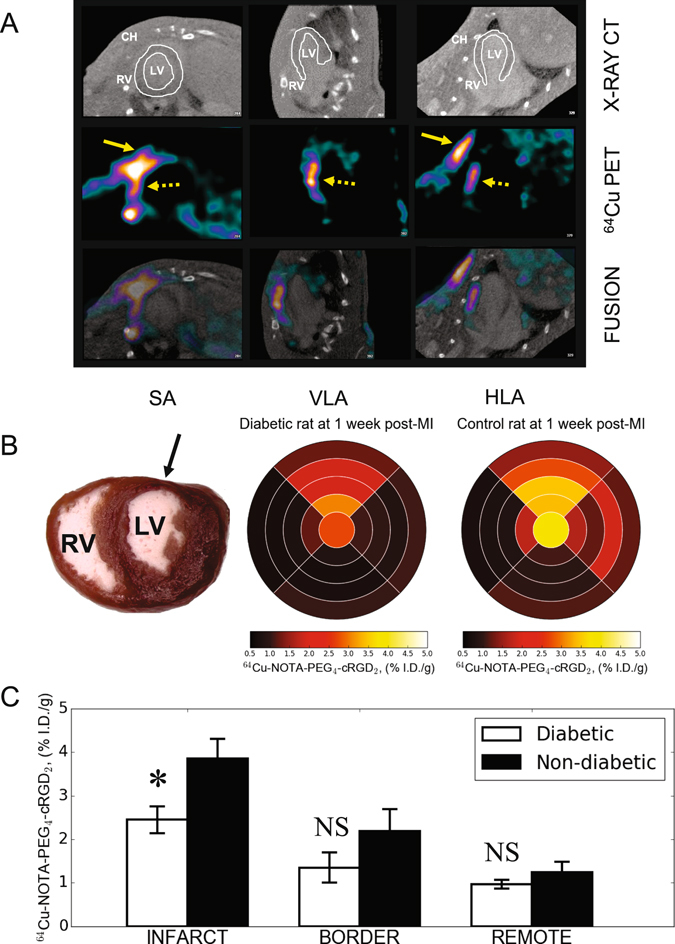
Representative *in vivo* hybrid PET-CT reconstructed short-axis (SA), vertical- (VLA) and horizontal long-axis (HLA) images acquired with iodinated contrast agent (Omnipaque) at 90 min post-injection of ^64^Cu-NOTA-PEG_4_-cRGD_2_ (**A**). The iodinated blood pool contrast agent permitted better definition of right (RV) and left ventricle (LV) within the myocardium which is contoured with solid white line. Focal uptake of ^64^Cu-NOTA-PEG_4_-cRGD_2_ was seen within anteriolateral LV regions (dashed yellow arrow) although significant uptake was seen in chest wall (CH) at the thoracotomy site (solid yellow arrows) indicating active wound healing associated *α*_*V*_ *β*_3_ receptor expression. Bull’s eye myocardial plots of ^64^Cu-NOTA-PEG_4_-cRGD_2_ activity in diabetic and non-diabetic Lewis rats subjected to surgical ligation of LAD to induce myocardial infarction (arrow) (**B**). Hearts were immediately excised at 120 min post-injection, cleaned and filled with inert dental molding material and cut in four 2-mm thick slices from apex to base. After removing right ventricle (RV), each left ventricular (LV) slice was cut in four segments (anterior, septal, posterior, lateral), and ^64^Cu radioactivity was measured in each segment with gamma well counting. Data for each slice were expressed as percentage of injected dose per gram tissue (%I.D./g) and categorized as infarct, border and remote areas (**C**).

To explore the feasibility of *in vivo* imaging and quantification of *α*_*V*_ *β*_3_ integrin activation in the myocardium, static *in vivo* PET-CT images were performed at 90 min after injection of (**7**) in both diabetic and non-diabetic control rats one week after myocardial infarction. To better define the right ventricular (RV) and left ventricular (LV) myocardium we administered the iodine-based X-ray contrast agent Omnipaque (GE Healthcare, USA) during CT acquisition. Representative co-registered PET-CT images are shown in Fig. 6A. PET images of (**7**) demonstrated strong focal uptake of the tracer in the infarcted area (dashed arrows), as well as in the chest wall at the site of the thoracotomy (solid arrows). The significant increase in (**7**) activity within infarcted myocardium that was observed by PET-CT imaging was confirmed by quantitative gamma well counting of myocardial sections. The retention of the radiotracer was expressed as %I.D./g tissue and graphed as circumferential Bull’s eye plots for myocardial slices spanning from the apex to the base, and divided into anterior, septal, posterior and lateral sections (Fig. 6B). LAD ligation resulted in anterior-lateral infarct, which was characterized by an increased (**7**) uptake. Radioactivity within the infarcted anterior sections was approximately 4-fold higher than in non-infarcted septal regions at one week after myocardial infarction (Fig. 6C).

### Post mortem evaluation of *α*_*V*_*β*_3_ expression in MI

**Figure 7:**
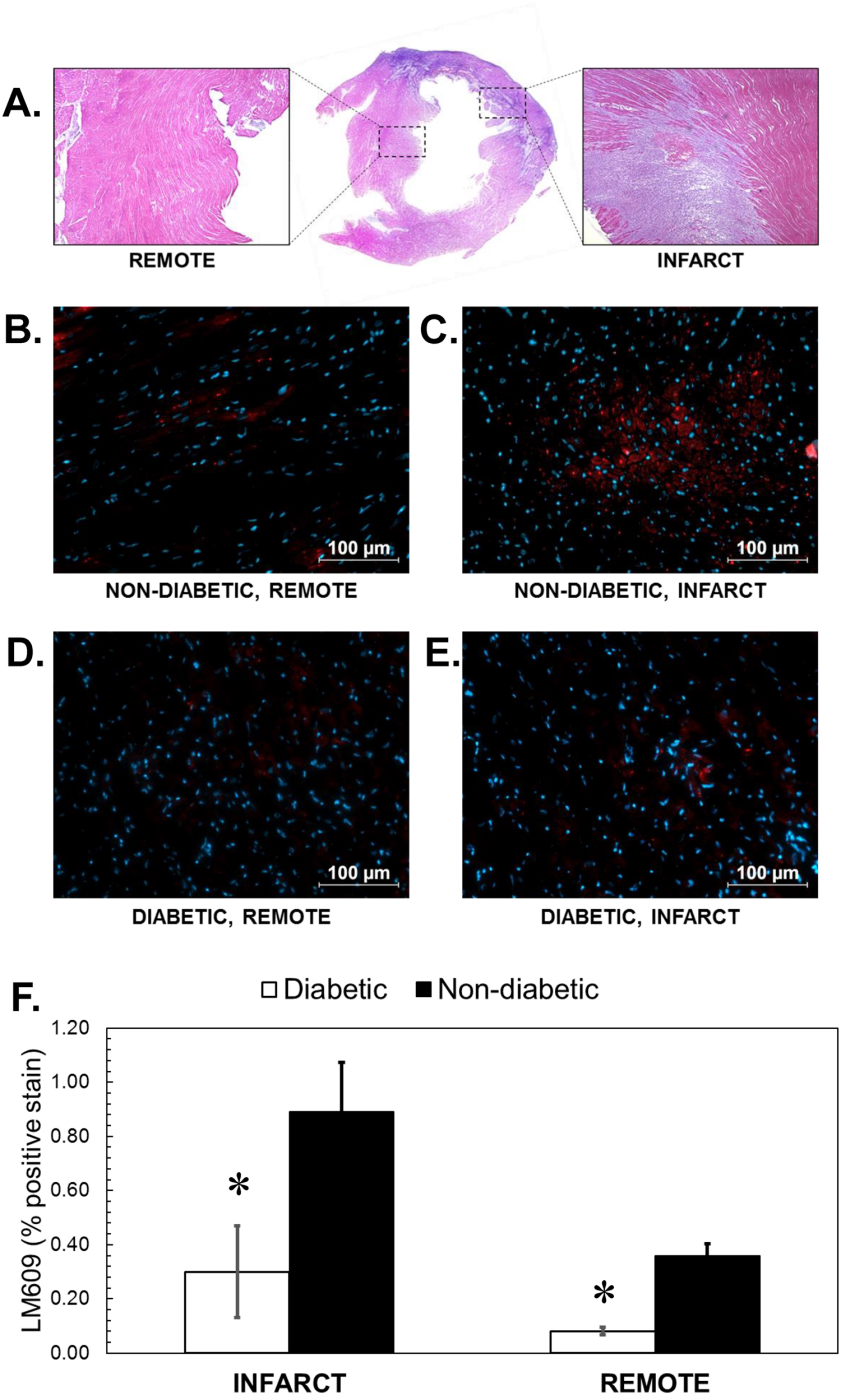
*α*_*V*_ *β*_3_ immunohistochemistry. (**A**) A representative H&E stain of the infarct and remote areas of the heart. (**B**–**E**) representative immunohistochemistry images with LM609 (red) and DAPI (blue). (**F**) Quantification of *α*_*V*_ *β*_3_ positive area in stained heart tissue. The non-DM infract tissue showed significant increase in *α*_*V*_ *β*_3_ expression relative to DM infract tissue.

To investigate *α*_*V*_ *β*_3_ expression at the cellular level, at one week post MI, a subset of animals was euthanized, sections of the heart muscle tissue were excised, and stained with a commercially available PE-LM609 (*α*_*V*_ *β*_3_ marker). Subsequent fluorescence imaging showed 2.4 fold decrease in the infract tissue of DM animals in comparison to the non-DM at the one week time point with little to none change in the remote area of both groups (Fig. 7). We have also demonstrated the same trend where diabetic animals had significantly reduced (~40%, *P < 0.05) myocardial uptake of (**7**) within the infarct, whereas there was no significant difference in both border and remote sections of diabetic and non-diabetic rats.

## Discussion

Dimers constitute a valuable subclass of RGD cyclopeptides, characterized by better targeting capability and higher integrin-specific uptake, when compared to their monocyclic counterparts.

In the past, a variety of linkers connecting two monomeric cyclic RGDs have been evaluated to modify radiotracer receptor binding affinity, organ uptake, biodistribution, and excretion kinetics from non-cancerous organs.

In this work, we describe the synthesis and chemical characterization of a novel dimeric cyclic-RGD probe for quantitative non-invasive imaging of *α*_*V*_ *β*_3_ activity followed by a multiscale biological analysis of probe’s receptor binding and its pharmacokinetics. In a series of experiments we explored probe’s *in vitro* binding affinity using surface plasmon resonance (SPR) with immobilized *α*_*V*_ *β*_3_ receptor and live endothelial cells expressing *α*_*V*_ *β*_3_. Finally, we evaluated the behavior of (**7**) *in vivo* and investigated its feasibility for non-invasive PET-CT imaging of *α*_*V*_ *β*_3_ expression following myocardial infarction in diabetic and non-diabetic animals.

We began our investigations by studying direct interactions between dimeric- and monomeric-cRGD probes and *α*_*V*_ *β*_3_ at the receptor level using surface plasmon resonance technique. SPR provides a powerful tool for the analysis of protein-protein and protein-ligand kinetics, including the determination of affinity, association and dissociation rates, and activation energies. SPR can be applicable even for low affinity systems which are often notoriously difficult to study using other techniques.

We demonstrated that our dimeric-cRGD construct is characterized by a very fast association to *α*_*V*_ *β*_3_ receptor (*k*_*on*_ = 1.1 × 10^7^ M^−1^ s^−1^) which is approximately 40-fold greater than that of previously characterized monomeric cRGDfK (*k*_*on*_ = 2.7 × 10^5^ M^−1^ s^−1^) see Table 1. In contrast, our receptor-bound dimeric-cRGD probe dissociated from the *α*_*V*_ *β*_3_ receptor at a much slower rate (*k*_*off*_ = 2.1 × 10^−6^ s^−1^) than classical adhesive proteins like fibrinogen and vibronectin (*k*_*off*_ = 9.8 × 10^−4^ s^−1^ and 2.1 × 10^−4^ s^−1^, respectively)^52^. All these results suggest that both dimerization and use of certain structural elements such as PEG linkers and 1,2,3-triazole moieties, contributed to improved association to *α*_*V*_ *β*_3_ integrin (hence longer bound lifetime), while leaving dissociation properties essentially unchanged.

To study probe-receptor interactions at the cellular level in a biologically relevant model we used human umbilical vein endothelial cells (HUVEC), which are known to constitutively express *α*_*V*_ *β*_3_ integrin.

The binding affinity (K_*d*_) of fluorescein (38.27 nM) and ^64^Cu -labeled (33.85 nM) dimeric cRGD probes were comparable to those of fibrinogen and vibronectin (27 nM and 64 nM, respectively). More importantly, the dissociation constant of our dimeric cRGD probes were approximately 20-fold smaller than most monomeric cRGD peptides, and similar to tetrameric cRGD probes reported in the literature^24^. Moreover, our dimeric-cRGD peptides have approximately only 2.5-fold lower affinity than *α*_*V*_ *β*_3_ integrin antibody (LM609, K_*d*_ = 14.4 nM), which suggests feasibility of (**7**) and (**8**) for high-sensitive targeted imaging of angiogenic activity. In addition, our tracer exhibits a nearly identical IC_50_ value (5.1 nM, see Fig. 4G) as that reported for ^18^F-Galacto-RGD (5 nM)^53^. While we have used endothelial cells in our own assay, we note that IC_50_ values can vary considerably from one cell-type to another^23^. This difference should be accounted for when comparing IC_50_ across cell lines.

Certain metal ions, when bound to the *β*_3_ subunit of *α*_*V*_ *β*_3_, are known to induce a conformational change that can affect the binding of cRGD ligands^54^. Manganese (II) ions, for example, activate the *α*_*V*_ *β*_3_ integrin, while calcium ions or strong chelators like EDTA lead to its deactivation. To study this phenomenon, we incubated HUVECs with Mn^2+^ or EDTA. The addition of manganese led to a dissociation constant that was considerably lower (13.8 nM) in comparison to excess EDTA (Fig. 4A,B). These results support the notion that metal ions like Mn^2+^ play an important role in mediating the interactions between *α*_*V*_ *β*_3_ integrin and cRGD, and illustrate the important role of environmental conditions when assessing the binding characteristics and potential of cRGD-based probes as imaging agents.

Angiogenesis is a key factor in the process of cardiac healing after myocardial ischemia. Much work was focused at targeting angiogenesis for both diagnosis and therapy. For this reason non-invasive methods for visualizing *α*_*V*_ *β*_3_ after myocardial infarction using both SPECT and PET have been reported in the literature. Noteworthy examples of radio-labeled *α*_*V*_ *β*_3_ probes include the ^111^In-labeled SPECT tracer ^111^In–RP748, Technetium-labeled SPECT tracers like ^99*m*^Tc-RAFT-RGD, ^99*m*^Tc-IDA-D-[c(RGDfK)]_2_, and ^99*m*^Tc-NC100692, as well as PET tracers such as ^18^F-Galacto-RGD, ^68^Ga-NODAGA-RGD, and the trimeric ^68^Ga-TRAP(RGD)_3_, all of which have been studied in animal models of myocardial infarction^55–59^. PET affords higher count numbers and sensitivity than SPECT (in part because PET does not use an extrinsic collimator), and as such our probe can be used to give more sensitive images with fewer artifacts than the available SPECT tracers. Moreover, our probe utilizes ^64^Cu because of its favorable decay characteristics (half life 12.7 h), which allow imaging at later time points than several other commonly used PET and SPECT isotopes like ^18^F, ^68^Ga and ^99*m*^Tc (half life 110 min, 68 min, and 6 h, respectively). While the rapid pharmacokinetics of our radiotracer may speak to using a short half-life radionuclide, we chose to employ a longer half-life radioisotope which enabled our (**7**) to be more broadly applicable: the required ^64^Cu can be produced at a nearby cyclotron facility and then used hours to a day later without requiring the type of on-site isotope production facilities that shorter-lived isotopes do. Moreover, the NOTA chelating moiety on our probe enabled fast single step labeling (~60 min) at room temperature. Other imaging probes can require higher temperatures and much long preparation times. The preparation of ^18^F-Galacto-RGD, for example, requires temperatures as high as 70 °C and takes approximately 200 minutes—significantly longer than the halflife of the ^18^F radionuclide itself^60^.

While several multimeric cRGD-based probes have been described in that past, it has been found that little sensitivity or specificity is gained by increasing the cRGD multiplicity past two cRGD units^56, 59^; as such, we have focused here on enhancing the pharmacokinetic properties of a dimeric cRDG probe. In designing the structure of our dimer we decided to employ two PEG linkers, and 1,2,3-triazole moieties. Combining both of these structural elements as pharmacokinetic modifiers was expected to improve the biodistribution and elimination of the tracer^49^. Additionally, we used NOTA when labeling with ^64^Cu, which has been shown to exhibit lower accumulation in the liver than other available bifunctional chelators such as DOTA^61^.

The favorable effects of these structural modifications on both pharmacokinetics and organ retention were verified with dynamic PET imaging followed by biodistribution studies, which provided vital insight into the *in vivo* behavior of (**7**). Within minutes after injection, we observed a rapid blood clearance of (**7**) with paralleled retention within kidney medulla and cortex. We also observed almost complete clearance after one hour with little gastrointestinal activity (Fig. 5), which we attributed to the hydrophilicity of (**7**). We found high kidney and bladder retention, but essentially no detectable radioactivity in feces (Figure SI 9), indicating the main excretion route is through kidney filtration and urine (which has also been observed in other cRGD-based radiotracers^31, 59^).

While several previous studies of myocardial infarction employed healthy animals, here we focused specifically on imaging myocardial infarction in diabetic rats. High resolution microPET-CT imaging using (**7**) in diabetic and non-diabetic rats subjected to LAD ligation and subsequent myocardial infarction demonstrated both discrete and focal myocardial distributions (Fig. 6). Our probe was found to localize to the infarcted tissue within the anterio-lateral region of the heart, and this *in vivo* imaging pattern was obtained consistently for different rats (both diabetic and non-diabetic) at 1 week after surgical induction of myocardial infarction. Importantly, we found that the ratio of probe uptake in the infarcted region of the heart compared to a normal region was significantly higher in non-DM rats than in DM rats (3.85 ± 0.51 versus 2.44 ± 0.35, respectively), indicating decreased *α*_*V*_ *β*_3_ activity and, in turn, decreased capacity for neovascularization in rats with diabetes. Similar infarcted vs. normal myocardial segments for healthy rats have been reported in prior studies (4.7 ± 0.8 using ^18^F-Galacto-RGD, 5.2 ± 0.8 using ^68^Ga-NODAGA-RGD, and 4.1 ± 0.7 using ^68^Ga-TRAP(RGD)_3_^55, 59^).

We found that the greatest *α*_*V*_ *β*_3_ activity occurred 1 week after induction of myocardial infarction in both healthy and diabetic rats, a finding in good agreement with previous studies using other RGD-based tracers in non-DM rats^58, 59^. This delayed *α*_*V*_ *β*_3_ uptake is a critical point to consider when designing potential stem-cell-based therapies for patients with myocardial infarction, especially those with other pathologies like diabetes that may limit their innate capacity for neovascularization. In particular, in diabetic patients, early detection and treatment could prove extremely important to myocardial infarction recovery, and to that end, rapid non-invasive imaging strategies like ours could play an important role in the clinical setting.

We demonstrated that *in vivo* targeted *α*_*V*_ *β*_3_ integrin imaging can be performed in combination with contrast CT imaging where the administration of X-ray contrast agent did improve the definition of the myocardial edges and the blood pool. These *in vivo* PET-CT images with contrast provided better localization of (**7**) to the site of the myocardial infarction within the anterior-lateral wall and allowed for differentiation between the myocardial wall and thoracotomy site, which was characterized by active wound healing associated *α*_*V*_ *β*_3_ integrin expression. Moreover, the use of the X-ray contrast agent permitted reconstruction of the *α*_*V*_ *β*_3_ images without the need for a reference perfusion image. Reconstruction and orientation of the PET images was extremely difficult without the reference CT images. The image properties of (**7**) are superior due to the low background activity.

This combination of both anatomical and molecular targeted imaging with dimeric-cRGD-based agents holds great promise for non-invasive quantitative assessment of different uptake patterns observed within the myocardium of diabetic and non-diabetic animals post-MI. Our results strongly suggest reduced *α*_*V*_ *β*_3_ integrin activation in the onset of diabetes. While the observed regional fluctuations in the radiotracer’s retention remain in general agreement with previously reported observations with monomeric cRGD based SPECT and PET imaging agents or more invasive non-imaging techniques^24, 62^, targeted molecular imaging with (**7**) demonstrated excellent quality images, higher or comparable specific uptake within infarcted tissue, and relatively low non-specific uptake in critical organs. All told, these imaging results clearly indicate that our dimeric-cRGD probe has a potential for quantitative mapping of spatial and temporal changes in *α*_*V*_ *β*_3_ activation in small animal models of myocardial injury in the onset of diabetes as well as potentially expanding these studies to larger animals and humans.

## Methods

### Synthesis and Chemical Characterization

The synthesis and chemical characterization (NMR and HR-MS) of both (**7**) and (**8**) have been described in detail in Supplementary data (Figures S1–S8).

### Ligand-Receptor Binding Kinetics Studies

All surface plasmon resonance (SPR) studies were performed with the BIAcore 3000 instrument (Biacore International AB, Uppsala, Sweden) at 25 °C on dextran-coated gold sensor chips (CM5, Research grade, GE Healthcare Bio-sciences AB, Uppsala) divided into four separate flow cells (three containing immobilized *α*_*V*_ *β*_3_ integrin receptor and one control). HBS-EP pH 7.4 (10 mmol HEPES, 3 mmol EDTA, 150 mmol NaCl, 0.002% TWEEN-20) was used as a running buffer.

Pre-concentration studies were performed to determine optimal pH conditions for receptor immobilization (Supplementary Table S1). Integrin *α*_*V*_ *β*_3_ receptor (R&D Systems, USA) was irreversibly immobilized to a single flow cell via amine coupling with the dextran matrix. A recombinant human CD-34 (R&D Systems, USA), a protein with no known interaction with peptides based on cRGD structure, was immobilized to a flow cell as a reference signal protein to account for non-specific interactions. Briefly, the surface was activated by injecting 35 *μ*L of 50 mM N-hydroxysuccinimide (NHS, GE Healthcare AB) and 200 mM EDC (GE Healthcare AB) at 1:1 ratio (v/v) and a flow rate of 5 *μ*L/min. Integrin *α*_*V*_ *β*_3_ receptor was dissolved at 20 *μ*g/mL in 10 mM acetate buffer at its optimal pH and injected at 5 *μ*L/min until the target surface immobilization level was reached (approximately 200–500 R.U. of immobilized receptor). After sufficient amount of protein was coupled, the surface was deactivated by injecting 35 *μ*L ethanolamine.

Kinetic studies with dimeric and monomeric cRGD ligands were performed on the target flow cell containing the immobilized integrin *α*_*V*_ *β*_3_ receptor and the reference flow cell simultaneously. 120 *μ*L of each ligand (10, 20, and 40 nmol in HBP-EP running buffer) was injected at 30 *μ*L/min through both flow cells, followed by a 10 minute running buffer injection to observe ligand-receptor dissociation. After recording SPR sensograms, a series of 5 *μ*L HCl (5 mM) and 5 *μ*L NaOH (10 mM) at 5 *μ*L/min was injected to remove any remaining bound ligand residues. This cycle was repeated twice for each concentration of the ligand.

The kinetic rate constants (association *k*_*on*_ and dissociation *k*_*off*_ constants), the goodness-of-fit parameter (*χ* ^2^) and the peak magnitude of the signal response (*R*_*max*_) were determined by performing global kinetic analysis on the binding curves for each ligand-receptor pair (Supplementary Data)^63–65^.

### Cell Culture

Human umbilical vein endothelial cells (HUVEC) were acquired from American Type Culture Collection (ATCC, USA) and grown to confluence in K-12 medium containing 10% heat-inactivated FBS (Invitrogen), 100 U/mL penicillin G, 100 *μ*g/mL streptomycin, 0.25 *μ*g/mL amphotericin B (HyClone) and were maintained in a humidified incubator at 37 °C with 5% CO_2_.

### Cellular Binding Studies

To investigate various cellular binding characteristics of radiolabeled (**7**) and its FITC-analogue, both gamma well counting and flow cytometry/fluorescence microscopy were used, respectively. HUVEC were incubated with (**7**) (0–100 nM) for 1 h at room temperature, filtered with 0.22 *μ*m centrifuge filters (Corning Incorporated, USA), washed twice with PBS (pH 7.4) and radioactivity measured with gamma well counter (Wizard2, Perkin-Elmer, USA). HUVEC were also incubated in either (**8**) or FITC-Galacto-cRGD_2_ (0–200 nM) for 1 h at room temperature. Cells were centrifuged, washed twice, and analyzed with a BD LSR II flow cytometer (BD Biosciences, USA) using gates set for FITC and corrected for auto-fluorescence. All experiments were performed in technical triplicates.

#### Integrin activation and deactivation

To study the effect of *α*_*V*_ *β*_3_ integrin activation/deactivation on cellular binding, HUVEC were pretreated with of Mn^2+^ or EDTA (20 *μ*M) for 10 min, washed with ice-cold PBS, and incubated with (**7**) (0–100 nM) or (**8**) (0–200 nM) for 1 h at room temperature. Cells were centrifuged, washed twice with PBS (pH 7.4), and analyzed with gamma well counter or flow cytometry using gates set for FITC and corrected for auto-fluorescence.

#### Competitive binding

HUVEC were pretreated with 50 *μ*M of unlabeled H-PEG_4_-cRGD_2_ for 30 min, and then incubated with (**7**) (0–100 nM) or (**8**) (0–200 nM) for 1 h at room temperature. Cells were centrifuged, filtered with 0.22 *μ*m centrifuge filters (Corning incorporated, USA), washed twice with PBS (pH 7.4), and analyzed with gamma well counter or flow cytometry using gates set for FITC and corrected for auto-fluorescence.

#### Competitive inhibition (IC_50_ determination)

HUVEC were incubated with 50 nM of (**7**) and increasing concentrations of H-PEG_4_-cRGD_2_ (0–1 *μ*M) for 1 h at room temperature, filtered, and washed twice with PBS (pH 7.4), and transferred to counting tubes for gamma well counting.

#### Specificity

In order to determine the specificity to *α*_*V*_ *β*_3_ integrin, HUVEC were incubated with (**8**) (0.34 *μ*M) and co-incubated with phycoerythrin (PE) conjugated anti- *α*_*V*_ *β*_3_ integrin antibody PE-LM609 (11.12 *μ*g/ml) (R&D Systems, USA) for 1 h at room temperature. Cells were centrifuged at 5000 rpm for 10 min and washed twice with ice-cold PBS (pH 7.4). Gates were set for PE, FITC, and corrected for auto-fluorescence and potential overlap between the PE and FITC channels.

#### Dual-fluorescence staining

HUVEC were grown to confluence on coverslips and fixed with 4% paraformaldehyde for dual-fluorescence staining with (**8**) (1 *μ*M) and anti- *α*_*V*_ *β*_3_ PE-LM609 antibody (1:100, R&D Systems, USA). The coverslips were incubated with each stain separately for 1 h at room temperature, with washing buffer (PBS, pH 7.4) rinses before and after each incubation for a duration of 5 min. Coverslips were mounted to slides with DAPI Fluoromount (Southern Biotech, USA), imaged with a confocal fluorescence inverted microscope (Zeiss LSM 700 Confocal, Germany) equipped with 63x oil objective, preprocessed with commercial software package (ZEN 2012, Zeiss, USA), and colocalization analyzed with Fiji software^66^.

### *In Vivo* Evaluation

#### Animal preparation

All *in vivo* imaging experiments were approved by the Institutional Animal Care and Use Committee of the University of Illinois at Urbana-Champaign, in accordance to the principles outlined by the American Physiological Society on research animal use.

#### Blood clearance, metabolism and biodistribution

To assess blood clearance and organ uptake dynamic *in vivo* PET-CT imaging was performed followed by image processing and analysis. Lewis rats (n = 6, Harlan Inc.) were anesthetized with 2% isoflurane, the neck area was shaved and a jugular vein cut-down procedure was performed to insert a PE-50 polyurethane catheter for radiotracer injection. Animals were then placed in supine position on the polyacrylic imaging bed of a small animal dedicated PET-CT scanner (Inveon, Siemens Healthcare, USA). At the time of PET acquisition, animals were injected with ~22.2 MBq of (**7**) via the jugular vein catheter over 30 s followed by bolus infusion of 0.2 mL sterile saline. Animals underwent 60 min dynamic PET imaging (15% energy window centered at 511 keV) followed by anatomical X-ray CT imaging (80 keV, 500 *μ*A, 100 *μ*m spatial resolution).

Following dynamic PET-CT imaging, both urine and feces samples were collected to assess radiotracer’s metabolism and excretion route. The urine samples were collected at 90 min post injection by manual void, mixed at 1:1 ratio (v/v) with 50% CH_3_ CN and centrifuged at 5000 rpm. The supernatant was collected, analyzed by HPLC (C18 column, flow 1 mL/min, CH_3_ OH/H_2_ O, 5/95, v/v), and chromatograms compared to reference samples of (**7**) and ^64^Cu-acetate. Feces samples were collected at 90 min post injection, suspended in 50% CH_3_ CN, homogenized and centrifuged at 5000 rpm. The supernatant was filtered and analyzed by RP-HPLC.

For biodistribution studies, all animals were euthanized at 90 min post-injection and selected organ samples were collected for gamma well counting analysis. All collected sections were weighed and the tissue radioactivity was measured with Wizard^2^ gamma well counter (Perkin-Elmer, USA). Measured ^64^Cu activity was corrected for background, decay time, and tissue weight.

#### *In vivo* imaging of myocardial angiogenesis

To evaluate the feasibility of (**7**) imaging in rodent model of myocardial ischemia, male diabetic and non-diabetic Lewis rats (n = 5 in each group) were subjected to surgical permanent ligation of left anterior descending (LAD) artery to induce anteriolateral myocardial infarction (MI) resulting in angiogenic response^62^. Type-1 diabetes was induced by bolus injection of 50 mg/kg streptozotocin (STZ), which resulted in hyperglycemia (>200 mg/dl glucose) and glucosuria at 6 weeks post STZ injection. To induce MI, animals were anesthetized with 1–3% isoflurane, and the respiration was controlled using an animal ventilator (Kent Scientific, USA) for a thoracotomy incision. Myocardial infarction was induced by ligating the proximal LAD coronary artery with 8–0 silk suture. One week after the surgery, all animals were anesthetized with 1–3% isoflurane, neck area shaved, and the left jugular vein was isolated for placement of a PE-50 polyurethane catheter to facilitate injection of the radiotracer. All animals were injected with ~29.6 MBq of (**7**). Imaging was performed using hybrid small animal microPET-CT scanner (Inveon, Siemens Healthcare, USA). Animals were placed on animal bed and ~60 min after radiotracer injection, a 15 min microPET imaging was performed. This was followed by a high-resolution anatomical microCT imaging (360 projections, 80 keV/500 *μ*A energy) during continuous (0.8 mL/min) intravenous infusion of an X-ray CT contrast agent (Omnipaque, GE Healthcare, USA) using programmable syringe pump (Kent Scientific, USA).

#### Image analysis and tissue processing

The microPET and microCT images were reconstructed using OSEM/3D iterative algorithm (Siemens Healthcare, USA) and cone-beam technique (Cobra Exim), respectively. Both microPET and microCT images were fused, reoriented and visualized as short (SA), vertical- (VLA) and horizontal-long (HLA) axis slices using Inveon Research Workplace (Siemens Healthcare, USA). Immediately after last imaging session, all animals were euthanized with intravenous injection of KCl (1 mol/L), hearts excised and washed in ice-cold buffered saline. Both right and left ventricles were filled with dental molding material to facilitate uniform cutting into 2 mm slices. All slices were then divided into four sections; anterior, lateral, posterior and septal. Collected sections were weighed and tissue radioactivity was measured with Wizard2 gamma well counter (Perkin-Elmer, USA). Measured ^64^Cu activity was corrected for background, decay time, and tissue weight, and expressed as percent injected dose (% I.D./g).

#### Histology and immunochemistry

A subset of the animals were euthanized at 1 week post surgery and their heart tissue were excised, embedded in TissueTec (Sakura, USA) and snap frozen in −150 °C methylbutane. Frozen sections (5 nm) were placed on microscope slides, fixed with pre-cooled acetone, and stored at −80 °C before staining. To assess heart muscle vascularity, heart samples were stained with an *α*_*V*_ *β*_3_ marker PE-LM609 Antibody (Abcam, USA). All staining procedures were performed according to the product-specific protocols (four for each slide). The stains were quantified for extent (percentage area) of positive staining in randomly chosen high-powered (200x) fields using algorithms validated by our group previously^57^.

## Conclusions

During the last decade, much effort has been expended on the chemical modification of RGD peptides to increase affinity for *α*_*V*_ *β*_3_ integrin. Recent studies suggested that cyclic RGD dimers have high affinity due to bivalency and have relatively low non-specific uptake in other critical organs. These properties can be further optimized by modification of the distance between the two RGD motifs using PEG linkers. It was also shown that introduction of 1,2,3-triazole moieties has a positive impact on the pharmacokinetic profile of receptor-binding ligands. All of this has been taken into consideration during the development of our dimeric cRGD probe. This study described the synthesis, chemical characterization, and multi-scale biological evaluation of both radiolabeled and fluorescent dimeric RGD peptide ligands for molecular imaging of *α*_*V*_ *β*_3_ integrin activation at the receptor, single cell, organ and whole-body level. Here we demonstrated that the targeted PET-CT imaging of regional activation of *α*_*V*_ *β*_3_ integrin within ischemic tissue holds the potential to directly quantify the extent and localization of ongoing angiogenic process *in vivo* and to assess this process on cellular level *in vitro* using optical imaging with a fluorescent analogue of the targeted imaging agent. The enhanced focal retention, favorable blood clearance kinetics and biodistribution, and excellent quality of images obtained with (**7**) suggest the potential for future clinical translation. This and other molecular imaging-based approaches should lead to better understanding of pathophysiology and development of novel paradigms for patient management.

## Electronic supplementary material

Supporting Information
