# Supplementary material for: Synthesis, Chemical Characterization and Multiscale Biological Evaluation of a Dimeric-cRGD Peptide for Targeted Imaging of αVβ3 Integrin Activity
Source: Sci Rep. 2017 Jun 9;7:3185. doi: 10.1038/s41598-017-03224-8 (PMC5466598; doi:10.1038/s41598-017-03224-8)
Supplement: Supplementary file 1 — Supporting Information [file 41598_2017_3224_MOESM1_ESM.pdf]

Synthesis, Chemical Characterization and Multiscale Biological Evaluation of a Dimeric-cRGD Peptide for Targeted Imaging of $\alpha_V\beta_3$ Integrin Activity

Jamila Hedhli[1,2,+], Andrzej Czerwinski[3,+], Matthew Schuelke[1,2], Agata Płoska[1,4], Paweł Sowinski[5], Lukas LaHood[1,2], Spencer B. Mamer[2], John A. Cole[6], Paulina Czaplewska[7], Maciej Banach[8], Iwona T. Dobrucki[1], Leszek Kalinowski[4], Princess Imoukhuede[2], and Lawrence W. Dobrucki[1,2,*]

[1]Beckman Institute for Advanced Science and Technology, Urbana, IL
[2]Department of Bioengineering, University of Illinois at Urbana-Champaign, Urbana, IL
[3]Peptides International Inc., Louisville, KY
[4]Department of Laboratory Diagnostics, Medical University of Gdansk, Poland
[5]NMR Laboratory, Faculty of Chemistry, Gdansk University of Technology, Poland
[6]Department of Physics, University of Illinois at Urbana-Champaign, Urbana, IL
[7]Intercollegiate Faculty of Biotechnology of the University of Gdansk and Medical University of Gdansk, Poland
[8]Department of Hypertension, Medical University of Lodz, Poland
[*]corresponding author: dobrucki@illinois.edu
[+]these authors contributed equally to this work

ABSTRACT

Cyclic peptides containing the Arg-Gly-Asp (RGD) sequence have been shown to specifically bind the angiogenesis biomarker $\alpha_V\beta_3$ integrin. We report the synthesis, chemical characterization, and biological evaluation of two novel dimeric cyclic RGD-based molecular probes for the targeted imaging of $\alpha_V\beta_3$ activity (a radiolabeled version, ^{64}Cu-NOTA-PEG$_4$-cRGD$_2$, for PET imaging, and a fluorescent version, FITC-PEG$_4$-cRGD$_2$, for *in vitro* work). We investigated the performance of this probe at the receptor, cell, organ, and whole-body levels, including its use to detect diabetes associated impairment of ischemia-induced myocardial angiogenesis. Both versions of the probe were found to be stable, demonstrated fast receptor association constants, and showed high specificity for $\alpha_V\beta_3$ in HUVECs ($K_d \sim 35$nM). Dynamic PET-CT imaging indicated rapid blood clearance via kidney filtration, and accumulation within $\alpha_V\beta_3$-positive infarcted myocardium. ^{64}Cu-NOTA-PEG$_4$-cRGD$_2$ demonstrated a favorable biodistribution, slow washout, and excellent performance with respect to the quality of the PET-CT images obtained. Importantly, the ratio of probe uptake in infarcted heart tissue compared to normal tissue was significantly higher in non-diabetic rats than in diabetic ones. Overall, our probes are promising agents for non-invasive quantitative imaging of $\alpha_V\beta_3$ expression, both *in vitro* and *in vivo*.

Supplementary Information

Synthesis and Chemical Characterization

Reagents Diphenylphosphoryl azide (DPPA), N-(3-dimethylaminopropyl)-N'-ethylcarbodiimide hydrochloride (EDC), N,N-diisopropylethylamine (DIPEA), triethylamine (TEA), copper(II) sulfate pentahydrate

($CuSO_4 \times 5H_2O$), sodium L-ascorbate, triisopropylsilane (TIPS) were purchased from Aldrich (St. Louis, MO). Boc-PEG$_4$-OH and isothiocyanatobenzyl-1,4,7- triazacyclononane- 1,4,7-triacetic acid (p-SCN-Bn-NOTA) were obtained from Quanta BioDesign (Plain City, OH) and Macrocyclics (Dallas, TX), respectively. Fluorescein isothiocyanate (5-FITC) was obtained from AnaSpec (Fremont, CA). 8-Azido-3,6-dioxaoctanoic acid (N_3-PEG$_2$-OH) and Boc-L-glutamic acid bis-propargyl amide were the products of Peptides International (Louisville, KY). Cyclo [Arg(Pbf)-Gly-Asp(OtBu)-D-Phe-Lys] was prepared according to the literature method[1].

Synthesis The synthesis of both ^{64}Cu-NOTA-PEG$_4$-cRGD$_2$ and FITC-PEG$_4$-cRGD$_2$ has been described in detail below:

(1) cyclo[Arg(Pbf)-Gly-Asp(OtBu)-D-Phe-Lys(PEG$_2$-N$_3$)] To a solution of cyclo [Arg(Pbf)-Gly-Asp(OtBu)-D-Phe-Lys] hydrochloride (1.71 g, 1.80 mmol) and N_3-PEG$_2$-OH (0.43 g, 2.27 mmol) in DMF (10 mL) was added DPPA (0.59 mL, 2.72 mmol), followed by the addition of Et_3N (0.50 mL, 3.59 mmol). The reaction mixture was kept at 0 °C for 5 h, with occasional adjustments of the pH value (7-8) with Et_3N. Then, the mixture was diluted with THF (100 mL), EtOAc (300 mL), and 0.4 N HCl (100 mL). The organic layer was washed with saturated $NaHCO_3$ (2 × 100 mL), brine (100 mL), dried over Na_2SO_4, and evaporated. The residue was triturated with isopropyl ether, and the product was collected by filtration, washed with the ether and dried under vacuum to afford 1.80 g of **(1)** in 92.3% yield as a colorless solid. ESI-MS: m/z = 1105.56 $[M+Na]^+$; 1083.51 $[M+H]^+$.

(2) Boc-Glucyclo[Arg(Pbf)-Gly-Asp(OtBu)-D-Phe-Lys(PEG$_2$-(1,2,3-triazole)-1-yl- 4-methylamide)]$_2$ To a stirred solution of (**1**) (1.765 g, 1.63 mmol) and Boc-L-glutamic acid bis-propargyl amide (0.253 g, 0.79 mmol) in a mixture of DMF (20 mL), t-BuOH (32 mL), and H_2O (11 mL) were added 0.2 M $CuSO_4$ x $5H_2O$ (2.6 mL) and 0.5 M sodium ascorbate solution (1 mL) at room temperature. After 1 h the reaction mixture was diluted with 1-BuOH (300 mL) and H_2O (200 mL). The organic layer was washed with H_2O (2 × 100 mL), and concentrated under reduced pressure. Addition of Et_2O resulted in a precipitate, which was collected by filtration, washed with Et_2O, and dried to give 1.95 g of (**2**) (99.6%).

(3) H-Glucyclo[Arg-Gly-Asp-D-Phe-Lys(PEG$_2$-(1,2,3-triazole)-1-yl- 4-methylamide)]$_2$ × 3 TFA The protected peptide (**2**) (1.94 g, 0.78 mmol) was dissolved in 20 mL of a solution TFA/TIPS/H_2O (95/2.5/2.5, v/v/v) at room temperature. After 2 h, the reaction mixture was concentrated under reduced pressure and the deprotected crude product was precipitated with cold Et_2O. It was collected by filtration, washed with Et_2O, and dried to give **(3)** in quantitative yield. ESI-MS: m/z = 591.07 $[M+3H]^{3+}$.

(4) Boc-PEG$_4$-Glucyclo[Arg-Gly-Asp-D-Phe-Lys(PEG$_2$-(1,2,3-triazole)-1-yl- 4-methylamide)]$_2$ A solution of Boc-PEG$_4$-OH (0.38 g, 1.04 mmol), N-hydroxysuccinimide (0.13 g, 1.13 mmol), and EDC hydrochloride (0.21 g, 1.10 mmol) in DMF (2 mL) was stirred at 0 °C. After 1 h, the ice bath was removed, and the reaction was allowed to proceed for 20 h at room temperature. The reaction mixture was then added to a stirred solution of **(3)** (1.65 g, 0.78 mmol) and DIPEA (0.73 mL, to adjust pH to 6) in DMF (15 mL). The reaction was allowed to proceed at room temperature for 6 h, with occasional adjustments of the pH value (7-8) with DIPEA. The reaction mixture was evaporated and the residue triturated with a mixture of acetonitrile and Et_2O. Precipitated peptide was collected by filtration, washed with Et_2O, and dried to yield **(4)** (1.85 g) as a crude product.

(5) H-PEG$_4$-Glucyclo[Arg-Gly-Asp-D-Phe-Lys (PEG$_2$-(1,2,3-triazole)-1-yl- 4-methylamide)]$_2$ × 3 TFA Boc-protected peptide **(4)** (1.85 g) was dissolved in 20 mL of TFA/H_2O (90/10, v/v) solution, and

the mixture was stirred at room temperature for 30 min. After evaporation of solvent, the residue was triturated with EtOAc and cold Et_2O. The crude product (1.50 g) was purified using preparative RP-HPLC to yield 735 mg of **(5)** as a white powder. The identity of the peptide was confirmed using HR-MS, monoisotopic mass calculated for $C_{88}H_{136}N_{28}O_{27}$: 2017.0128 m/z, $[M+H]^+$: 2018.0202; found: $[M+3H]^{+3}$ 673.3440 m/z, corresponding to molecular mass 2017.0079, accuracy 2.4 ppm.

(6) NOTA-PEG$_4$-cRGD$_2$ $\times$ 2TFA Compound **(6)** was prepared in the reaction of peptide **(5)** (200 mg, 0.085 mmol) with p-SCN-Bn-NOTA $\times$ 3 HCl (50 mg, 0.089 mmol) in DMF (5.7 mL) in a presence of DIPEA used for adjustment of pH to 8 (room temperature, 20 h). The product was precipitated with EtOAc and purified using preparative RP-HPLC to yield the title compound as a trifluoroacetate salt (57 mg, 24.9%) with a purity of 95.5% (Figure 1). The peptide was characterized by NMR (Tables 2 and 3) and HR-MS, m/z calculated for $C_{108}H_{162}N_{32}O_{33}S$ average mass: 2468.7361, monoisotopic mass: 2467.1702, $[M+H]^+$ 2468.1775; found: $[M+4H]^{+4}$ 617.7966 m/z, and monoisotopic $[M+3H]^{+3}$ 823.4048 m/z, which corresponds to molecular mass 2467.1724, accuracy 0.9 ppm.

(7) ^{64}Cu-NOTA-PEG$_4$-cRGD$_2$ Compound **(6)** was dissolved in 0.5 mL ammonium acetate buffer (100 mmol, pH 5.5) at room temperature. To the solution, about 37 MBq of Copper-64 (^{64}Cu) acetate in 0.02 mL ammonium acetate buffer (100 mmol, pH 5.5) was added. Radiolabeling was completed at room temperature after 30 min incubation and was confirmed by RP-HPLC (Agilent C18 column, flow 1 ml/min, CH_3OH/H_2O, 5/95, v/v) and thin-layer chromatography (Scan-RAM, LabLogic). For all experiments, radiochemical purity (% RCP) was at least 95%.

(8) Synthesis of FITC-PEG$_4$-cRGD$_2$ Compound **(8)** was prepared in the reaction of peptide **(5)** (300 mg, 0.127 mmol) with fluorescein-5-isothiocyanate (54.4 mg, 0.140 mmol) in DMF (4 mL) in a presence of DIPEA used for adjustment of pH to 8 (room temperature, 4 h). The peptide was precipitated with Et_2O and purified using preparative RP-HPLC to give 241 mg (71.9%) of the desired product as a trifluoroacetate salt with a purity of 97.7%. The structure of the peptide FITC derivative was verified using HR-MS, m/z calculated for $C_{109}H_{147}N_{29}O_{32}S$ average mass: 2407.6084, monoisotopic mass: 2406.0486, $[M+H]^+$: 2407.0559; found: $[M+4H]^{+4}$ 602.5226 m/z, and monoisotopic $[M+3H]^{+3}$ 803.0215 m/z, which corresponds to molecular mass 2406.0495, accuracy 0.4 ppm.

Characterization NMR spectra were recorded with a Varian Unity 500 spectrometer (Palo Alto, CA) in DMSO-d6/TFA. 2D NMR techniques including DQF-COSY, NOESY, TOCSY, gHSQC and gHMBC were used to confirm the structure of the synthesized NOTA-PEG$_4$-cRGD$_2$. Preparative RP-HPLC purifications were performed using a Shimadzu preparative system consisting of two LC-8A pumps controlled by a Shimadzu SCL-10A system controller, and a Shimadzu UV/VIS detector SPD-6A (λ=220 nm) equipped with a preparative flow cell. A Prochrom preparative column (50 mm x 35 cm) packed with Luna C18 (100Å pore size, particle size 10 μm) from Phenomenex (Torrance, CA) was used for the separations at a flow rate of 100 mL/min. Analytical HPLC runs were performed on Jupiter (Phenomenex) C18 column (4.6 mm x 250, pore size 300Å, particle size 5 μm) with a flow rate of 1 mL/min. Peptides were eluted using a linear gradient of 0.05% trifluoroacetic acid (TFA) in CH_3CN (Solvent B) and 0.05% aqueous TFA (Solvent A). High-resolution (HR) mass spectra (MS) were recorded on HR electrospray mass spectrometer (Waters Synapt G2-Si ESI MS). MS experimental conditions were as follows: the capillary voltage 3.5 kV, sample cone 25 V, source offset 80, source temperature 100 °C, and desolvation temperature 150 °C, cone gas nitrogen 5 L/h, desolvation gas nitrogen 800 L/h, nebuliser gas nitrogen 6.5 bar. Whole samples were run by flow injection, and the mobile phase was CH_3CN/water (1:1, v/v) at a flow rate of 100 μl/min.

Within NOTA fragment all carbon resonances were distinguishable, however direct labeling of few protons and carbon signals by N5, N6, N8, N9, N1a, N7b and N1b, N4b, N7b was not possible. Also, due to the compound symmetry, the two identical cRGD fragments cannot be differentiated; however, their integration in comparison to well separated H-E3 and H-E4 protons could serve as proof of their presence. The amino acid sequence was established based on the NOE's NH/CHα and the H/C NH/C=O/CHα correlation.

H and C atoms of the PEG and triazole fragments between lysine and glutamic acid, were assigned upon series of H/C and NOE correlations, namely: H-K6/C-13/H-12/C-10/H-10 and H-6/H-9, H-6/H-7, H-4/H-6, H-4'/H-6, H-2/H-4, H-2'/H-4', H-2/H-1/H-E2, H-2'/H-1'/H-E4. Similarly, bonds between glutamic acid and PEG as well as between benzyl groups and NOTA were established.

Ligand-Receptor Kinetics Studies

All ligand-receptor kinetics studies were performed with surface plasmon resonance (SPR) using the BIAcore 3000 instrument (Biacore International AB, Uppsala, Sweden).

Protein immobilization Pre-concentration studies were performed to determine optimal pH conditions for protein immobilization. This helped to ensure that the target levels of immobilized protein could be achieved precisely while also enabling us to conserve materials. Receptor solutions were prepared at 20 μg/mL with acetate buffer (10 mmol, pH ranging from 0.5 to 3.5, at -1.0 below the protein's isoelectric point, Table 1). Each receptor solution (20 μL) was injected at a flow rate of 5 μL/min, followed by a 5 μL injection of ethanolamine-HCL (GE Healthcare AB, Uppsala, Sweden) to clear out the binding surface of the receptor. The optimal pH of acetate buffer for each protein was selected according to the maximum level of protein immobilization reached in the pre-concentration study sensograms.

Ligand-receptor kinetics measurements The kinetic rate constants were determined by performing global kinetic analysis on the binding curves for each ligand-receptor pair with the BIAevaluation software (Version 4.1.1, GE Healthcare). In global kinetic analysis, nonlinear least squares was used to determine the association and dissociation constants that produced the best fit for multiple response curves, simultaneously. Global analysis is considered to produce the most accurate results in comparison to fitting of a single response curve[2]. Raw sensogram were processed for a given ligand-receptor pair. Briefly, each raw response curve was aligned and subtracted from the reference cell signal of each receptor signal to remove the effects of non-specific interactions. Once processed, a 1:1 Langmuir binding model (Equation 1) was fit to the data, and the association (k_{on}) and dissociation (k_{off}) kinetic constants, as well as the goodness-of-fit parameter (χ^2) and the peak magnitude of the signal response (R_{max}) were determined. This analysis was applied to each set of kinetic studies, except where the subtracted association phase was negative. A negative association curve occurs when the non-specific binding events on the reference cell exceed the binding events observed for the actual ligand-receptor pair; hence, it is indicative of a non-interaction.

$$L + R \underset{k_{off}}{\overset{k_{on}}{\rightleftharpoons}} LR \tag{1}$$

Next, the χ^2-to-R_{max} ratios were calculated which provide a well-established heuristic to determine the validity of obtained parameters for each ligand-receptor kinetic study. Previous studies have suggested that (χ^2) value obtained by fitting kinetic constants globally should be at least 10% of, or lower than, the signal's R_{max} (the change in signal from the highest level to the baseline)[3,4].

References

1. **1.** Haubner, R. *et al.* [18f]galacto-rgd: synthesis, radiolabeling, metabolic stability, and radiation dose estimates. *Bioconjug Chem* **15**, 61–9; DOI: 10.1021/bc034170n (2004). URL: https://www.ncbi.nlm.nih.gov/pubmed/14733584.
2. **2.** Myszka, D. G. Kinetic analysis of macromolecular interactions using surface plasmon resonance biosensors. *Curr Opin Biotechnol* **8**, 50–7; DOI: 10.1016/S0958-1669(97)80157-7 (1997). URL: https://www.ncbi.nlm.nih.gov/pubmed/9013659.
3. **3.** Biacore, A. *BIAevaluation Software Handbook 4* (1997).
4. **4.** Karlsson, R. Affinity analysis of non-steady-state data obtained under mass transport limited conditions using biacore technology. *J Mol Recognit* **12**, 285–92; DOI: 10.1002/(SICI)1099-1352(199909/10)12:5<285::AID-JMR469>3.0.CO;2-Y (1999). URL: https://www.ncbi.nlm.nih.gov/pubmed/10556876.

Supplementary Tables

Protein	pI	Optimal pH
Anti-hCD34	8.7	6.0
$\alpha_V \beta_3$	5.6	4.0

Table 1. Isoelectric points (pI) and optimal immobilization pHs for immobilized proteins.

loc	nr	group	δ (ppm)
Glu	E2	CH	4.22
	E2a	NH	8.00
	E3	CH_2	1.70, 1.88
	E4	CH_2	2.09, 2.12
triazole	1/1′	2 × NH	8.33/8.26
	2/2′	2 × CH_2	4.28/4.26
	4/4′	2 × CH	7.82/7.85
PEG	6	2 × CH_2	4.47
	7	2 × CH_2	3.79
	9,10	4 × CH_2	3.53
	12	2 × CH_2	3.81
Lys	K2	2 × CH	3.90
	K2a	2 × NH	8.02
	K3	2 × CH_2	1.39, 1.52
	K4	2 × CH_2	0.99
	K5	2 × CH_2	1.29
	K6	2 × CH_2	3.00
	K6a	2 × NH	7.62
Arg	R2	2 × CH	4.14
	R2a	2 × NH	7.58
	R3	2 × CH_2	1.45, 1.69
	R4	2 × CH_2	1.35
	R5	2 × CH_2	3.08
	R6	2 × NH	7.48
	R7a, R8	$2 \times (2 \times NH_2)^+$	6.80, 7.20
Gly	G2	2 × CH_2	3.23, 4.02
	G2a	2 × NH	8.35
Asp	D2	2 × CH_2	2.37, 2.69
	D3	2 × CH	4.62
	D3a	2 × NH	8.06
Phe	F2	2 × CH	4.43
	F2a	2 × NH	7.96
	F3	2 × CH_2	2.80, 2.89
	FPh	10 × CH	7.10, 7.16, 7.22
PEG	2″	CH_2	2.38
	3″	CH_2	3.55
	5″, 6″, 11″, 12″	CH_2	3.46
	8″, 9″, 14″	CH_2	3.52
	15″	CH_2	3.62
	16″	NH	7.69
	18″	NH	9.60
NOTA	Bn2, Bn3	4 × CH	7.18, 7.40
	Bn4a	CH_2	2.51, 2.80
	N2	CH	3.33
	N3	CH_2	2.97, 3.04
	N	7 × CH_2	2.7–4.0

Table 2. ^1H NMR data of NOTA-PEG_4-$cRGD_2$ in DMSO-d6/TFA

loc	nr	group	δ (ppm)
Glu	E1	C=O	171.4
	E2	CH	52.2
	E3	CH_2	28.2
	E4	CH_2	31.7
	E5	C=O	171.4
triazole	$2/2'$	CH_2	34.3
	$3/3'$	C=C	144.8
	$4/4'$	=CH	123.0
PEG	6	CH_2	49.3
	7	CH_2	68.6
	9,10	$2 \times CH_2$	68.3
	12	CH_2	70.1
	13	C=O	169.1
Lys	K1	C=O	172.2
	K2	CH	54.5
	K3	CH_2	30.7
	K4	CH_2	22.7
	K5	CH_2	28.7
Arg	R1	CH	171.3
	R2	C=O	51.9
	R3	CH_2	28.7
	R4	CH_2	25.2
	R5	CH_2	40.3
	R7	C=N	156.6
Gly	G1	CH_2	169.6
	G2	C=O	43.2
Asp	D1	CH	171.7
	D2	C=O	34.9
	D3	CH_2	48.4
	D4	C=O	170.1
Phe	F1	CH	170.7
	F2	C=O	54.3
	F3	CH_2	37.3
	FPh	C_6H_5	126.2, 128.2, 129.1, 137.3
PEG	$1''$	C=O	170.3
	$2''$	CH_2	35.8
	$3''$	CH_2	66.6
	$5''$, $6''$, $11''$, $12''$	CH_2	68.9–70.3
	$8''$, $9''$, $14''$	CH_2	68.3–70.3
	$15''$	CH_2	43.4
NOTA	Bn	C_6H_4	122.7,129.1,133.9,137.7
	Bn4a	CH_2	33.4
	N2	CH	58.6
	N3	CH_2	52.4
	N	CH_2	42.9,44.5,50.8,51.5,53.2,54.2,54.2
	N	COOH	168.8, 171.6, 173.4

Table 3. ^{13}C NMR data of NOTA-PEG$_4$-cRGD$_2$ in DMSO-d6/TFA (upon gHSQC and gHMBC spectra $\pm$ 0.2 ppm)

Supplementary Figures

N_3-PEG_2-OH + cyclo[Arg(Pbf)-Gly-Asp(O*t*Bu)-D-Phe-Lys] x HCl

↓ **DPPA/DMF/TEA**

cyclo[Arg(Pbf)-Gly-Asp(O*t*Bu)-D-Phe-Lys(PEG_2-N_3)] **(1)**

↓ **Boc-L-glutamic acid bis-propargyl amide**
$CuSO_4$ x $5H_2O$, sodium L-ascorbate
DMF/*t*-BuOH/H_2O

Boc-Glu{cyclo[Arg(Pbf)-Gly-Asp(O*t*Bu)-D-Phe-Lys(PEG_2-(1,2,3-triazole)-1-yl-4-methylamide)]}$_2$ **(2)**

↓ **TFA/TIPS/H_2O**

H-Glu{cyclo[Arg-Gly-Asp-D-Phe-Lys(PEG_2-(1,2,3-triazole)-1-yl-4-methylamide)]}$_2$ **(3)**

↓ **Boc-PEG_4-OSu**
DMF/DIPEA

Boc-PEG_4-Glu{cyclo[Arg-Gly-Asp-D-Phe-Lys(PEG_2-(1,2,3-triazole)-1-yl-4-methylamide)]}$_2$ **(4)**

↓ **TFA/H_2O**

H-PEG_4-Glu{cyclo[Arg-Gly-Asp-D-Phe-Lys(PEG_2-(1,2,3-triazole)-1-yl-4-methylamide)]}$_2$ **(5)**

↓ **p-SCN-Bn-NOTA**
DMF/DIPEA

NOTA-PEG_4-Glu{cyclo[Arg-Gly-Asp-D-Phe-Lys(PEG_2-(1,2,3-triazole)-1-yl-4-methylamide)]}$_2$ **(6)**

↓ **$^{64}CuCl_2$**
ammonium acetate buffer pH5

^{64}Cu-NOTA-PEG_4-Glu{cyclo[Arg-Gly-Asp-D-Phe-Lys(PEG_2-(1,2,3-triazole)-1-yl-4-methylamide)]}$_2$ **(7)**

Figure 1. Detailed synthesis of ^{64}Cu-NOTA-PEG_4-cRGD$_2$.

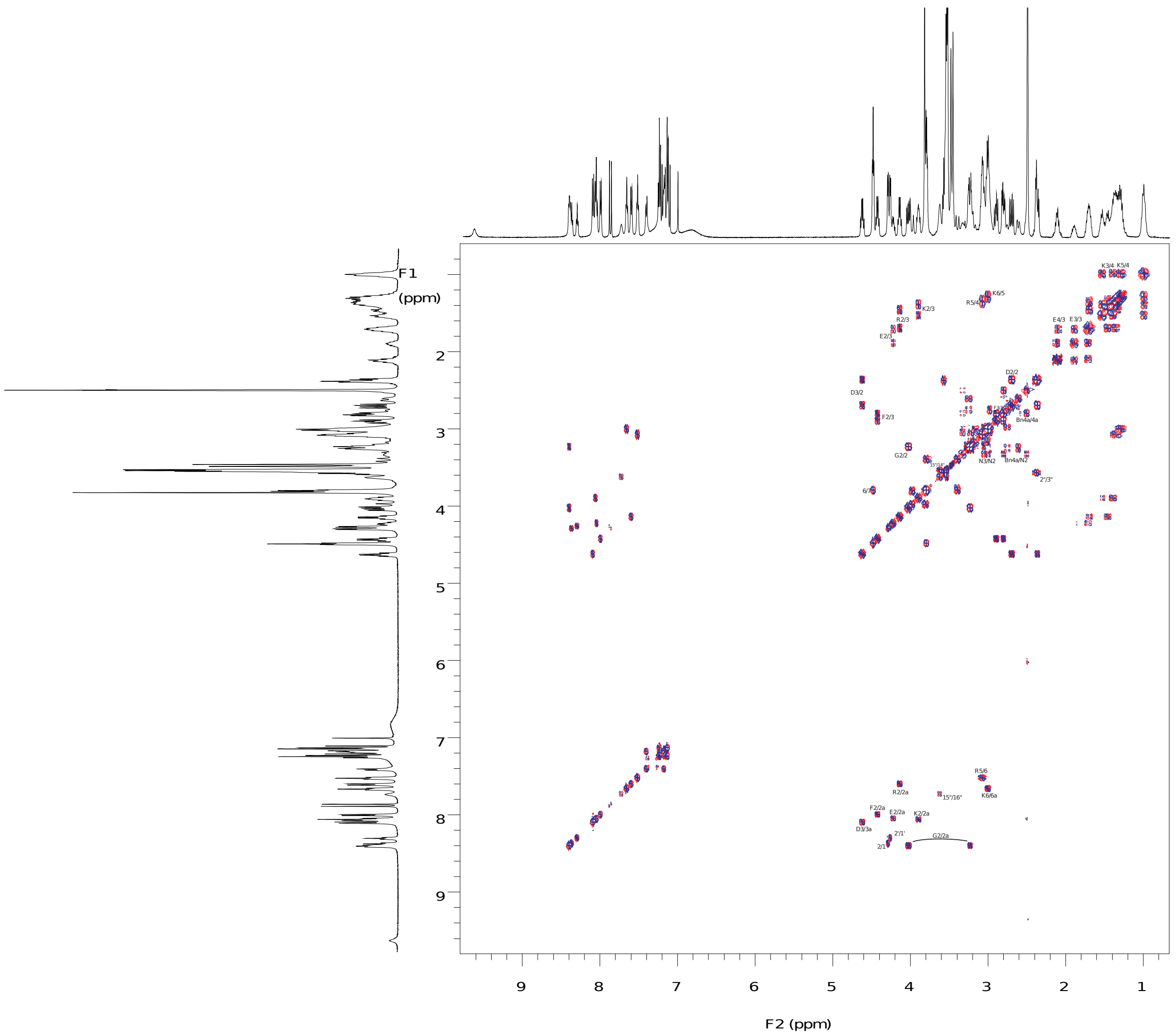

Figure 2. DQF-COSY spectrum of NOTA-PEG$_4$-cRGD$_2$ in DMSO-d6/TFA solution.

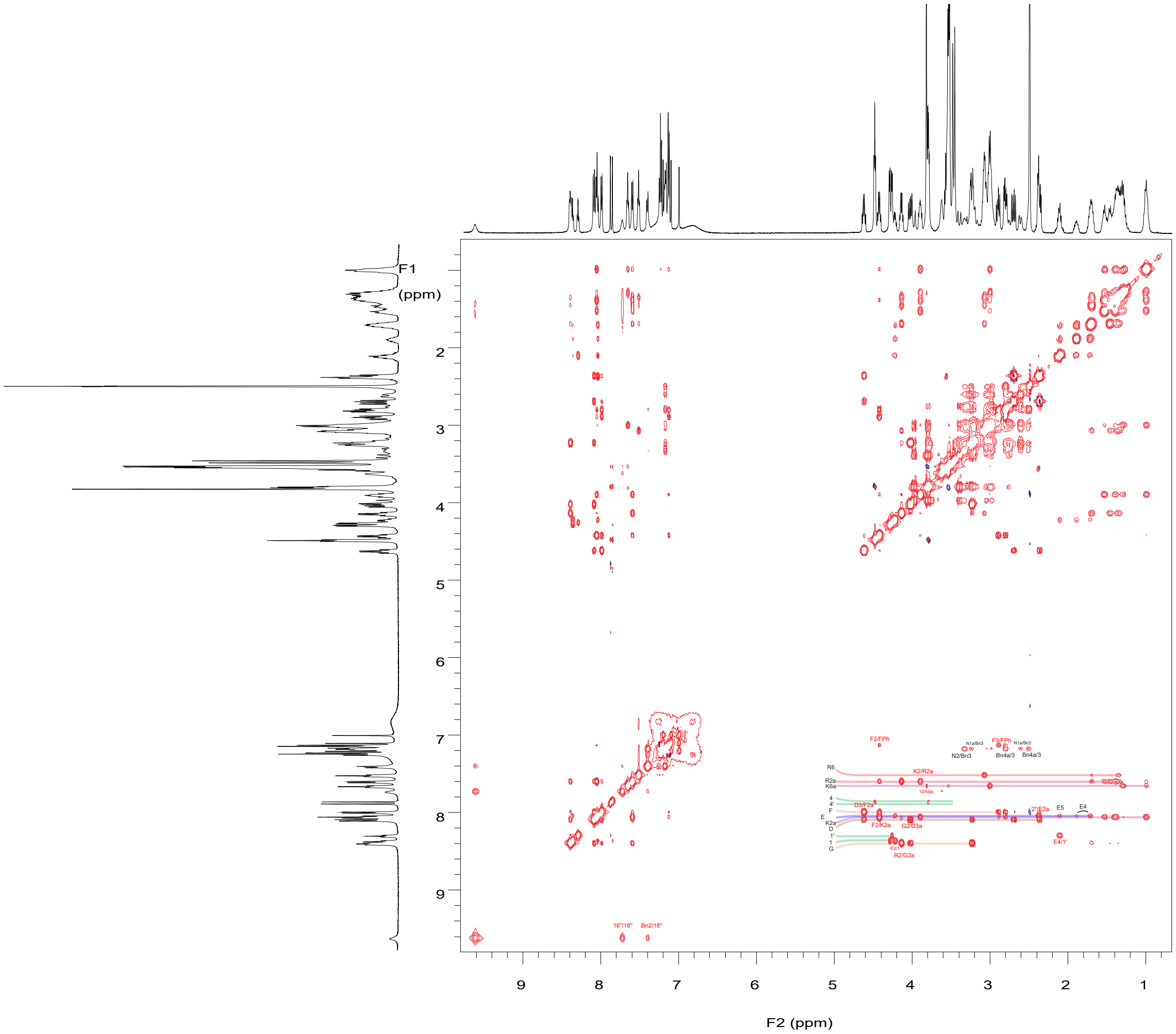

Figure 3. NOESY spectrum of NOTA-PEG4-cRGD2 in DMSO-d6/TFA solution.

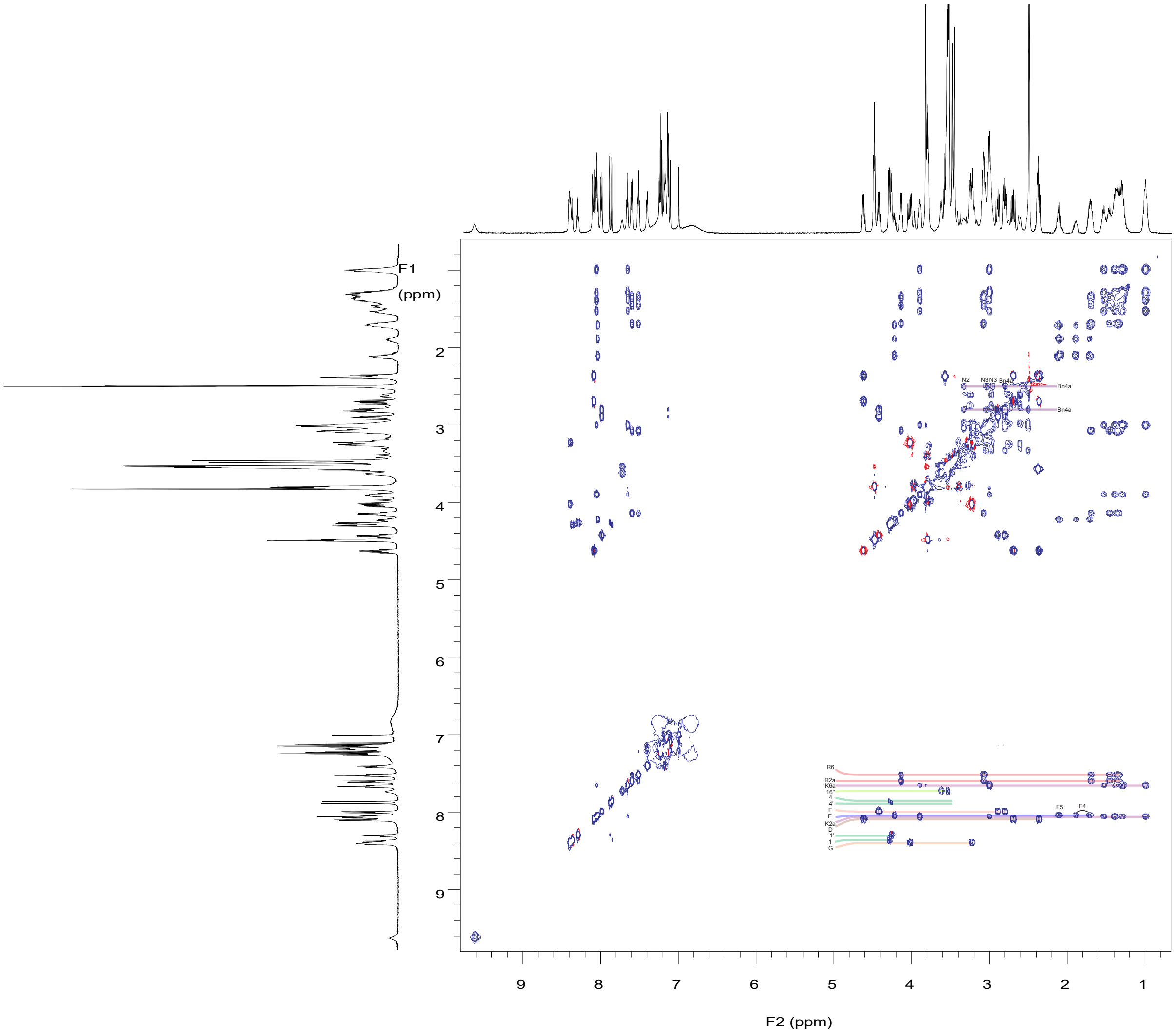

Figure 4. TOCSY spectrum of NOTA-PEG$_4$-cRGD$_2$ in DMSO-d6/TFA solution.

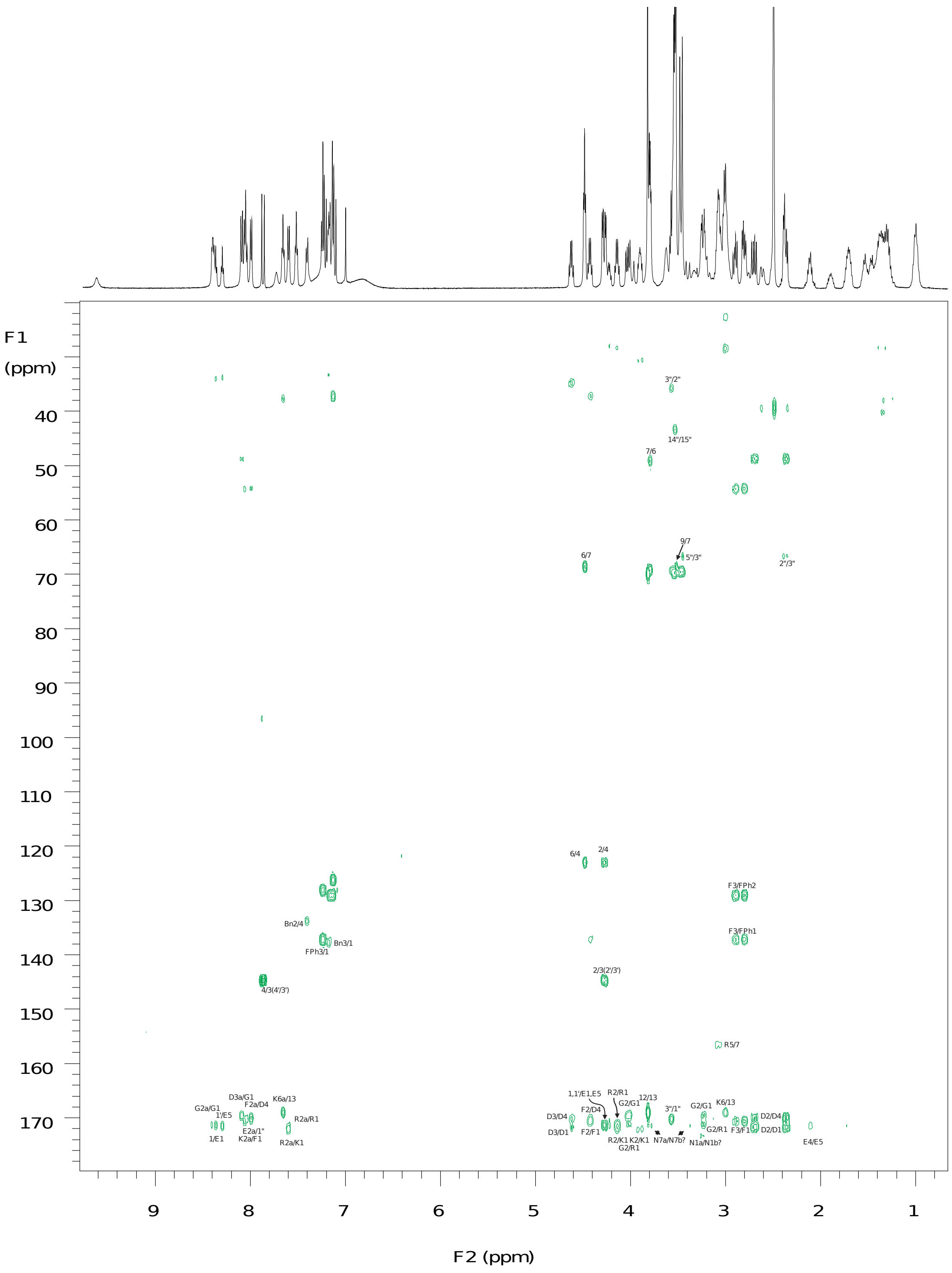

Figure 5. gHMBC spectrum of NOTA-PEG$_4$-cRGD$_2$ in DMSO-d6/TFA solution.

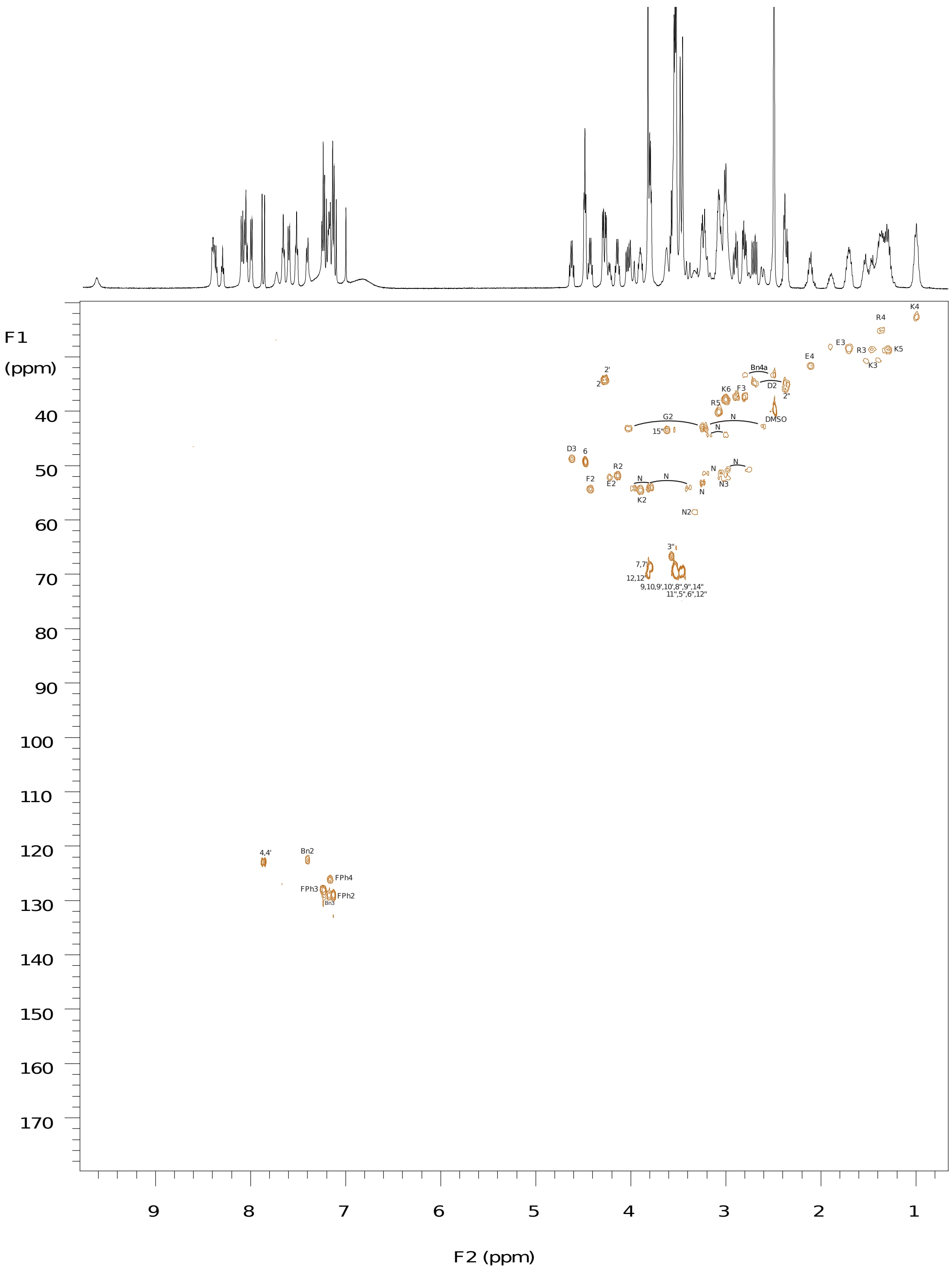

Figure 6. gHSQC spectrum of NOTA-PEG$_4$-cRGD$_2$ in DMSO-d6/TFA solution

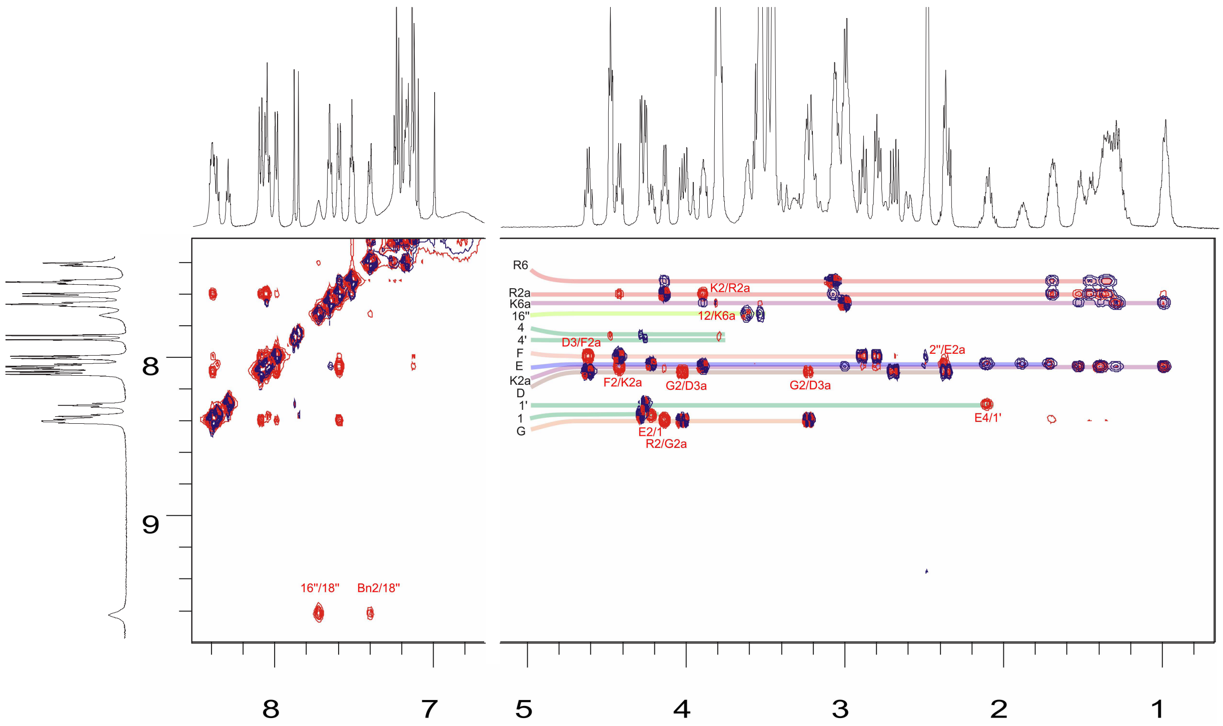

Figure 7. Superposition of TOCSY, DQF-COSY and NOESY NMR spectra of NOTA-PEG$_4$-cRGD$_2$ connectivities through amide protons.

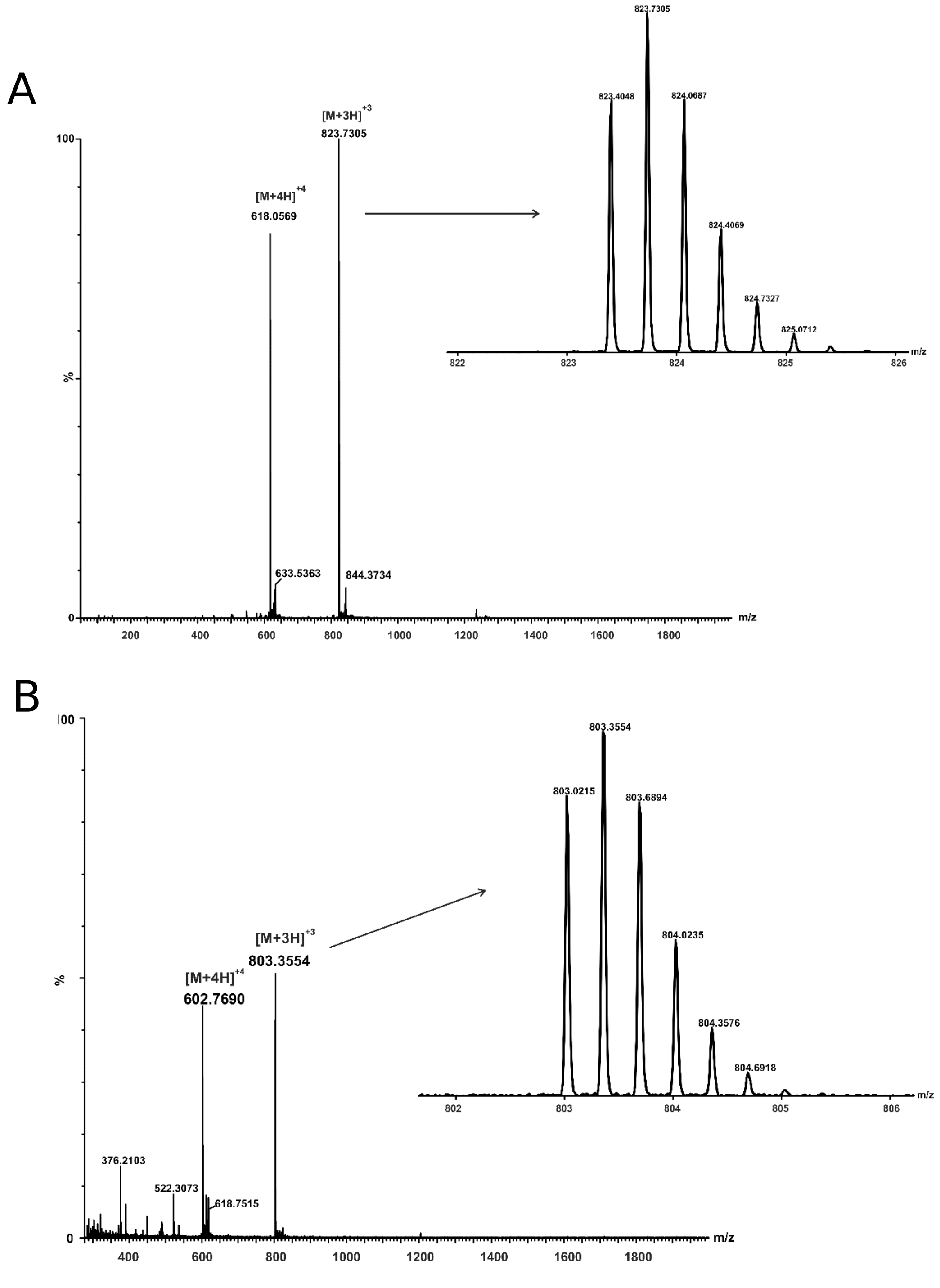

Figure 8. ESI mass spectra of (A) NOTA-PEG$_4$-cRGD$_2$ ($[M+3H]^{+3}$ m/z = 823.7305, $[M+H]^{+4}$ m/z=618.0569) zoom in on triply charged ion revealed monoisotopic signal m/z=823.4048; (B) FITC-PEG$_4$-cRGD$_2$ ($[M+3H]^{+3}$ m/z = 803.3554, $[M+H]^{+4}$ m/z=602.7690) zoom in on $[M+3H]^{+3}$ ion revealed triply charged monoisotopic signal m/z=823.4048

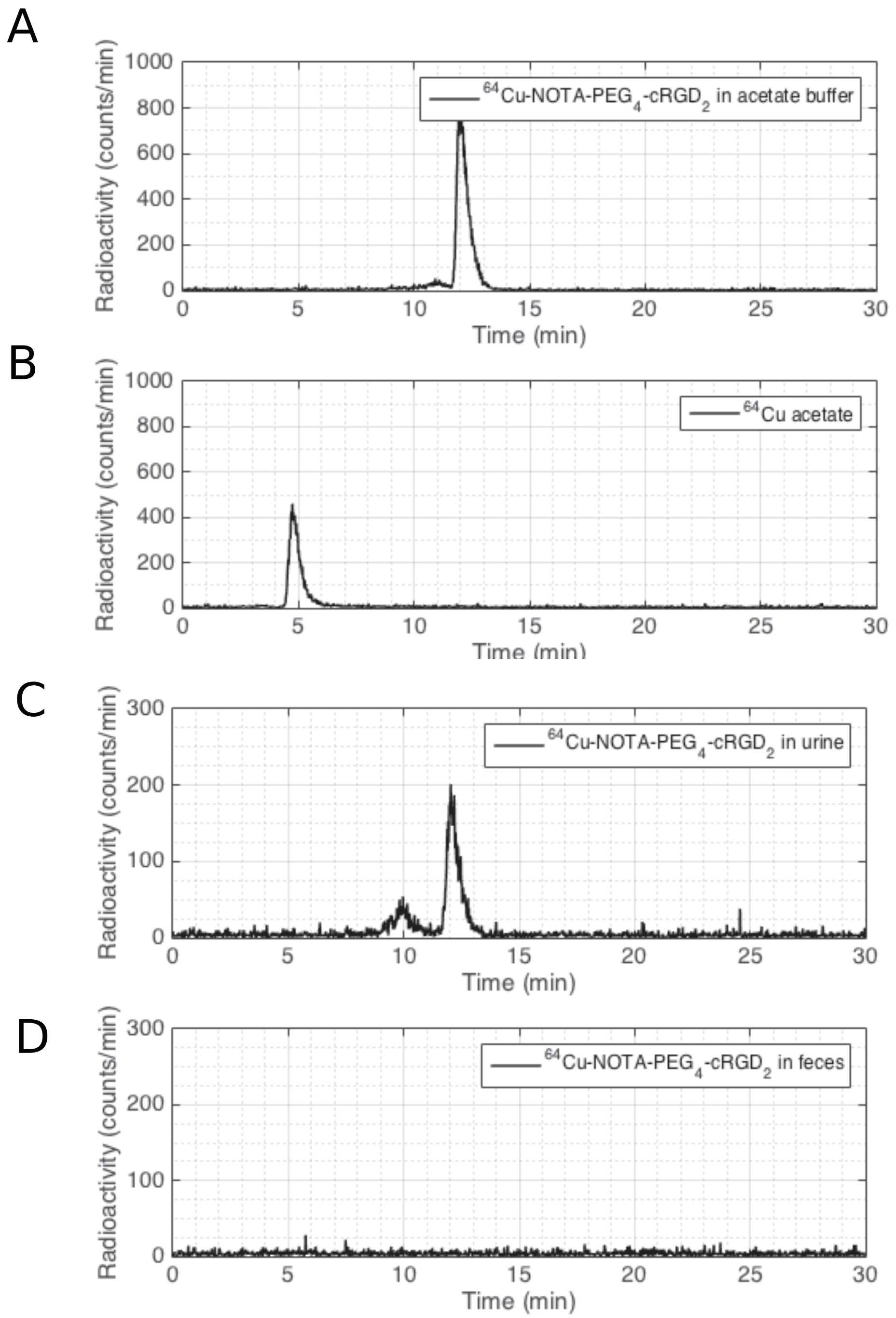

Figure 9. Representative radio-HPLC chromatograms for (A) ^{64}Cu-NOTA-PEG$_4$-cRGD$_2$ in acetate buffer 30 min after radiolabeling and immediately before injection, (B) ^{64}Cu-acetate used for radiolabeling, (C) ^{64}Cu-NOTA-PEG$_4$-cRGD$_2$ in urine at 90 min post-injection, and (D) ^{64}Cu-NOTA-PEG$_4$-cRGD$_2$ in feces at 90 min post-injection.
